# The selective autophagy receptors Optineurin and p62 are both required for zebrafish host resistance to mycobacterial infection

**DOI:** 10.1371/journal.ppat.1007329

**Published:** 2019-02-28

**Authors:** Rui Zhang, Monica Varela, Wies Vallentgoed, Gabriel Forn-Cuni, Michiel van der Vaart, Annemarie H. Meijer

**Affiliations:** Institute of Biology Leiden, Leiden University, Leiden, The Netherlands; McGill UniversityHealth Centre, CANADA

## Abstract

Mycobacterial pathogens are the causative agents of chronic infectious diseases like tuberculosis and leprosy. Autophagy has recently emerged as an innate mechanism for defense against these intracellular pathogens. *In vitro* studies have shown that mycobacteria escaping from phagosomes into the cytosol are ubiquitinated and targeted by selective autophagy receptors. However, there is currently no *in vivo* evidence for the role of selective autophagy receptors in defense against mycobacteria, and the importance of autophagy in control of mycobacterial diseases remains controversial. Here we have used *Mycobacterium marinum* (Mm), which causes a tuberculosis-like disease in zebrafish, to investigate the function of two selective autophagy receptors, Optineurin (Optn) and SQSTM1 (p62), in host defense against a mycobacterial pathogen. To visualize the autophagy response to Mm *in vivo*, *optn* and *p62* zebrafish mutant lines were generated in the background of a GFP-Lc3 autophagy reporter line. We found that loss-of-function mutation of *optn* or *p62* reduces autophagic targeting of Mm, and increases susceptibility of the zebrafish host to Mm infection. Transient knockdown studies confirmed the requirement of both selective autophagy receptors for host resistance against Mm infection. For gain-of-function analysis, we overexpressed *optn* or *p62* by mRNA injection and found this to increase the levels of GFP-Lc3 puncta in association with Mm and to reduce the Mm infection burden. Taken together, our results demonstrate that both Optn and p62 are required for autophagic host defense against mycobacterial infection and support that protection against tuberculosis disease may be achieved by therapeutic strategies that enhance selective autophagy.

## Author summary

Tuberculosis is a serious infectious disease that claims over a million lives annually. Vaccination provides insufficient protection and the causative bacterial pathogen, *Mycobacterium tuberculosis*, is becoming increasingly resistant to antibiotic therapy. Therefore, there is an urgent need for novel therapeutic strategies. Besides searches for new antibiotics, considerable efforts are made to identify drugs that improve the immune defenses of the infected host. One host defense pathway under investigation for therapeutic targeting is autophagy, a cellular housekeeping mechanism that can direct intracellular bacteria to degradation. However, evidence for the anti-mycobacterial function of autophagy is largely based on studies in cultured cells. Therefore, we set out to investigate anti-mycobacterial autophagy using zebrafish embryos, which develop hallmarks of tuberculosis following infection with *Mycobacterium marinum*. Using red-fluorescent mycobacteria and a green-fluorescent zebrafish autophagy reporter we could visualize the anti-mycobacterial autophagy response in a living host. We generated mutant and knockdown zebrafish for two selective autophagy receptors, Optineurin and p62, and found that these have reduced anti-bacterial autophagy and are more susceptible to infection. Moreover, we found that increased expression of these receptors enhances anti-bacterial autophagy and protects against infection. These results provide new evidence for the host-protective function of selective autophagy in mycobacterial disease.

## Introduction

Autophagy is a fundamental cellular pathway in eukaryotes that functions to maintain homeostasis by degradation of cytoplasmic contents in lysosomes [1]. During autophagy, protein aggregates or defective organelles are sequestered by double-membrane structures, called isolation membranes or phagophores, which mature into autophagosomes capable of fusing with lysosomes. Autophagy was previously considered a strictly non-selective bulk degradation pathway. However, recent comprehensive studies have highlighted its selective ability. Selective autophagy depends on receptors that interact simultaneously with the cytoplasmic material and with the autophagosome marker microtubule-associated protein 1 light chain 3 (Lc3), thereby physically linking the cargo with the autophagy compartment [2, 3]. Different selective autophagy pathways are classified according to their specific cargo; for example, mitophagy is the pathway that degrades mitochondria, aggrephagy targets misfolded proteins or damaged organelles, and xenophagy is directed against intracellular microorganisms. Recent studies have firmly established xenophagy as an effector arm of the innate immune system [4–6]. The xenophagy pathway targets microbial invaders upon their escape from phagosomes into the cytosol, where they are coated by ubiquitin. These ubiquitinated microbes are then recognized by selective autophagy receptors of the Sequestosome 1 (p62/SQSTM1)-like receptor (SLR) family, including p62, Optineurin (OPTN), NDP52, NBRC1, and TAX1BP1 [5]. In addition to targeting microbes to autophagy, SLRs also deliver ubiquitinated proteins to the same compartments. It has been shown that the processing of these proteins into neo-antimicrobial peptides is important for elimination of the pathogen *Mycobacterium tuberculosis* in macrophages [7].

*M*. *tuberculosis* (Mtb) is the causative agent of chronic and acute tuberculosis (Tb) infections that remain a formidable threat to global health, since approximately one-third of the human population carry latent infections and 9 million new cases of active disease manifest annually. Current therapeutic interventions are complicated by increased incidence of multi-antibiotic resistance of Mtb and co-infections with Human Immunodeficiency Virus (HIV). Despite decades of extensive research efforts, the mechanisms of how Mtb subverts the host’s innate immune defenses are incompletely understood, which poses a bottleneck for developing novel therapeutic strategies [8]. Because of the discovery of autophagy as an innate host defense mechanism, the potential of autophagy-inducing drugs as adjunctive therapy for Tb is now being explored [9].

Many studies have shown that induction of autophagy in macrophages by starvation, interferon-γ (IFN-γ) treatment, or by autophagy-inducing drugs, promotes maturation of mycobacteria-containing phagosomes and increases lysosome-mediated bacterial killing [7, 10–12]. Furthermore, it has been shown that the ubiquitin ligase Parkin and the ubiquitin-recognizing SLRs p62 and NDP52 are activated by the escape of Mtb from phagosomes into the cytosol [13, 14]. Subsequently, the ubiquitin-mediated xenophagy pathway targets Mtb to autophagosomes [13, 14]. Parkin-deficient mice are extremely vulnerable to Mtb infection [14]. However, a recent study has questioned the function of autophagy in the host immune response against Mtb, since mutations in several autophagy proteins, with the exception of ATG5, did not affect the susceptibility of mice to acute Mtb infection [15]. The susceptibility of ATG5-deficient mice in this study was attributed to the ability of ATG5 to prevent a neutrophil-mediated immunopathological response rather than to direct autophagic elimination of Mtb. In the same study, loss of p62 did not affect the susceptibility of mice to Tb, despite that p62 has previously been shown to be required for autophagic control of Mtb in macrophages [7, 15]. These different reports suggest that Mtb is able to suppress autophagic defense mechanisms and that the host requires strong autophagy induction to overcome such bacterial virulence mechanisms [12]. Taken together, the role that autophagy plays in Tb is complex and further studies are required to determine if pharmacological intervention in this process is useful for a more effective control of this disease.

In this study, we utilized zebrafish embryos and larvae to investigate the role of selective autophagy during the early stages of mycobacterial infection, prior to the activation of adaptive immunity. Zebrafish is a well-established animal model for Tb that has generated important insights into host and bacterial factors determining the disease outcome [16, 17]. Infection of zebrafish embryos with *Mycobacterium marinum* (Mm), a pathogen that shares the majority of its virulence factors with Mtb, results in the formation of granulomatous aggregates of infected macrophages, considered as a pathological hallmark of Tb [17–19]. Using a combination of confocal imaging in GFP-Lc3 transgenic zebrafish larvae and correlative light and transmission electron microscopy, we have previously shown that double membrane autophagic vesicles capture single Mm bacteria and fuse with larger Mm-containing degradative compartments [20, 21]. Furthermore, we found that the DNA-damage regulated autophagy modulator Dram1 is upregulated during infection by the central Myd88-NFκB innate immunity signaling pathway and protects the zebrafish host against Mm infection by a p62-dependent mechanism [21]. However, the role of p62 and other SLRs in host defense against Mm remains to be further elucidated.

p62 is known to function cooperatively with OPTN in xenophagy of *Salmonella enterica* [22–24]. Both these SLRs are phosphorylated by Tank-binding kinase 1 (TBK1) and bind to different microdomains of ubiquitinated bacteria as well as interacting with Lc3 [23, 25]. While several studies have implicated p62 in autophagic defense against Mtb, OPTN has thus far not been linked to control of mycobacterial infection [7, 13, 24–26]. We found gene expression of *p62* and *optn* to be coordinately upregulated during granuloma formation in zebrafish larvae [27], and set out to study the function of these SLRs by CRISPR/Cas9-mediated mutagenesis. We found that either p62 or Optn deficiency increased the susceptibility of zebrafish embryos to Mm infection, while overexpression of *p62* or *optn* mRNAs enhanced Lc3 association with Mm and had a host-protective effect. These results provide new *in vivo* evidence for the role of selective autophagy as an innate host defense mechanism against mycobacterial infection.

## Results

### Mm bacteria are ubiquitinated during infection of zebrafish

**Fig 1:**
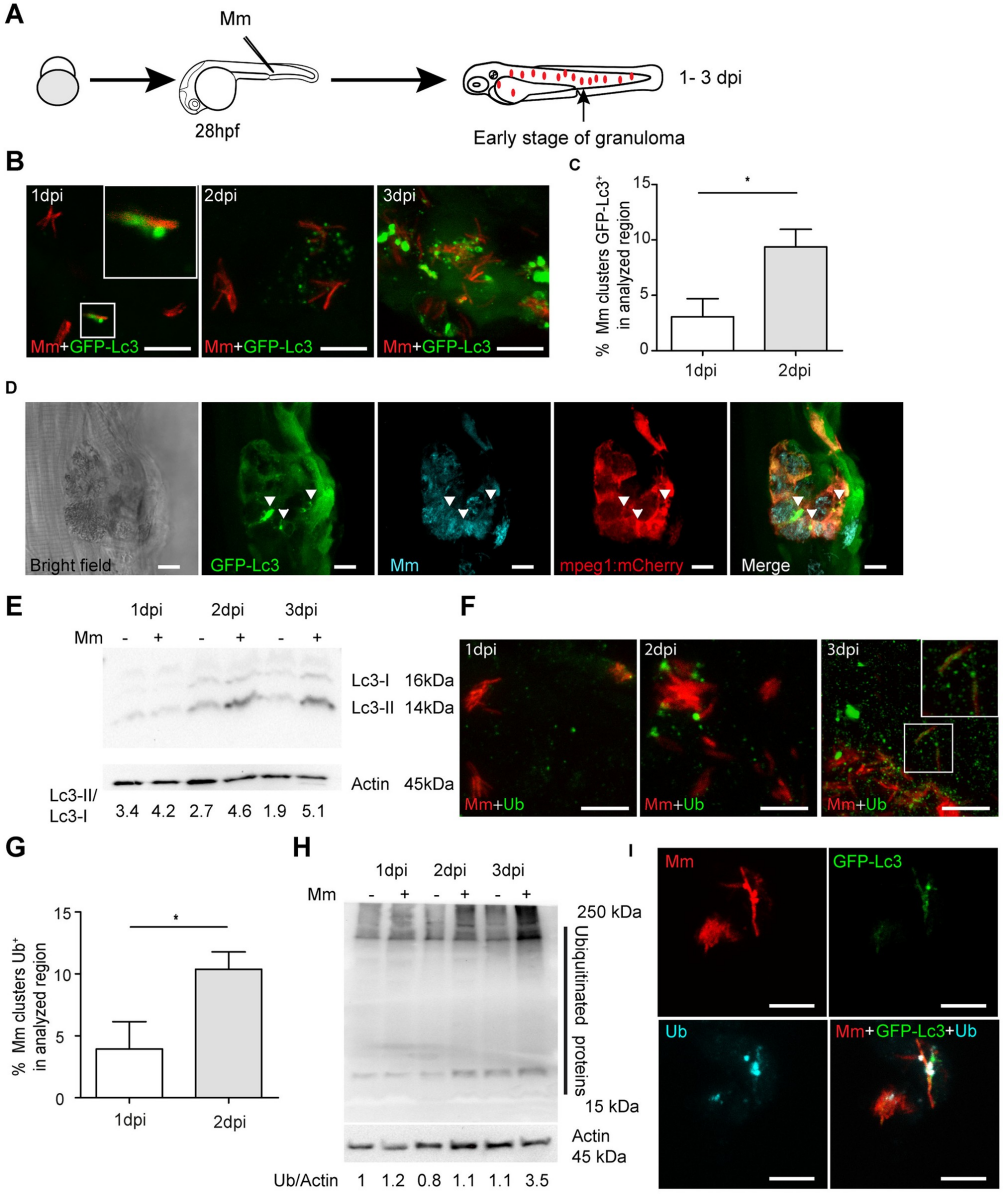
Ubiquitination and autophagy activity can be induced by Mm infection. (A) Schematic diagram of the zebrafish Mm infection model for early stages of Tb. Fluorescently labelled Mm bacteria are microinjected into the blood island of embryos at 28 hpf. Red dots represent small clusters of Mm-infected cells visible from 1 dpi. At 3 dpi these Mm clusters have grown into early stage granulomas. (B) Representative confocal micrographs of GFP-Lc3 co-localization with mCherry-labeled Mm clusters in infected embryos/larvae at 1, 2 and 3 dpi. Scale bars, 10 μm. (C) Quantification of the percentage of Mm clusters positive for GFP-Lc3 at 1 and 2 dpi. The results were accumulated from two individual experiments (≥ 20 embryos/group). ns, non-significant, *p<0.05, **p<0.01, ***p<0.001. (D) Representative confocal micrographs of GFP-Lc3 co-localization with mCrimson-labeled Mm clusters in an mpeg1:mCherry-expressing macrophage at 3 dpi. Arrowheads indicate intra-macrophage GFP-Lc3-positive Mm. Scale bars, 10 μm. (E) Western blot determination of Lc3 protein levels in Mm-infected embryos/larvae and uninfected controls. Protein samples were extracted at 1, 2 and 3 dpi (>10 larvae/sample). The Western blot was probed with antibodies against Lc3 and Actin as a loading control and is representative for three independent experimental repeats. The Lc3-II/Lc3-I ratio is indicated below. (F) Representative confocal micrographs of Ubiquitin antibody co-localization with Mm clusters in infected embryos/larvae at 1, 2 and 3 dpi. Scale bars, 10 μm. (G) Quantification of the percentage of Mm clusters positive for ubiquitin staining at 1 and 2 dpi (≥ 15 embryo/group). The results are accumulated from two independent experiments. ns, non-significant, *p<0.05, **p<0.01, ***p<0.001. (H) Western blot analysis of ubiquitination levels in Mm-infected embryos/larvae and uninfected controls. Protein samples were extracted at 1, 2 and 3 dpi (>10 larvae/sample). The Western blot was probed with an antibody detecting both poly- and mono-ubiquitin and with anti-Actin antibody as a loading control and is representative for three independent experimental repeats. (I) Representative confocal micrographs of GFP-Lc3 and Ubiquitin co-localization with Mm clusters in infected larvae at 3 dpi. Scale bars, 10 μm.

Phagosomal permeabilization and cytosolic escape of Mtb is known to induce the STING-dependent DNA-sensing pathway, resulting in ubiquitination and targeting of bacteria to autophagy [13]. We have previously shown that this pathway is also functional in zebrafish larvae infected with Mm and that a failure to induce autophagy reduces host resistance [21]. However, it had not been formally demonstrated that Mm bacteria are ubiquitinated in this model. To examine whether ubiquitin interacts with Mm and Lc3 during infection of zebrafish, we infected embryos at 28 hours post fertilization (hpf) and performed immunostaining for ubiquitin at 1, 2, and 3 days post infection (dpi), time points at which the early stages of tuberculous granuloma formation can be observed (Fig 1A). This process of granuloma formation is known to be induced by infected macrophages, which attract new macrophages that subsequently also become infected [28]. Developing granulomas also attract neutrophils and usually contain extracellular bacteria released by dying cells [29]. Using the zebrafish Tg(*CMV*:*GFP-map1lc3b*) autophagy reporter line [30], hereafter referred to as GFP-Lc3, we observed that around 3% and 9% of mCherry-labelled Mm clusters are targeted by GFP-Lc3 at 1 and 2 dpi, respectively, which increases to non-quantifiable levels at 3 dpi because of the increasing numbers and size of granulomas (Fig 1B and 1C). Mm is known to be rapidly phagocytosed after intravenous infection and replicate inside zebrafish macrophages [31, 32]. To confirm that the GFP-Lc3 targeting of Mm clusters occurs in macrophages, we used mCrimson-labelled Mm to infect double transgenic embryos with the GFP-Lc3 marker and the macrophage-specific and membrane-localized *mpeg1*:*mCherryF* marker [33] (Fig 1D). The results of GFP-Lc3 imaging were confirmed by Western blot, showing that LC3-II protein levels–indicative of autophagosome formation–gradually increased during Mm infection compared to uninfected controls (Fig 1E). Using a FK2 ubiquitin antibody, which can recognize monoubiquitinated cell surface molecules as well as polyubiquitin chains, we observed that ubiquitin co-colocalized with approximately 4% and 10% of the Mm clusters at 1 and 2 dpi, respectively (Fig 1F and 1G). Ubiquitin staining strongly increased by 3 dpi, not only in association with Mm clusters but also in the surrounding tissue (Fig 1F). In agreement, we observed by Western blot detection that Mm infection increased general levels of protein ubiquitination at 3 dpi (Fig 1H). In addition, we found that ubiquitin and GFP-Lc3 co-localized at Mm clusters (Fig 1I). Ubiquitin has been found to associate with cytosolic bacteria as well as with damaged membranes of phagosomes [34]. While we could not distinguish between these possibilities, we observed several instances of a tight association between ubiquitin signal and Mm bacteria, suggestive of direct ubiquitination of the bacteria (Fig 1F (inset) and Fig 1I). Collectively, these data demonstrate that Mm clusters are marked by ubiquitin and that overall ubiquitination levels are increased during infection in the zebrafish model, which coincides with autophagic targeting of bacteria.

### Deficiency in the ubiquitin receptors Optn or p62 does not impair zebrafish development

**Fig 2:**
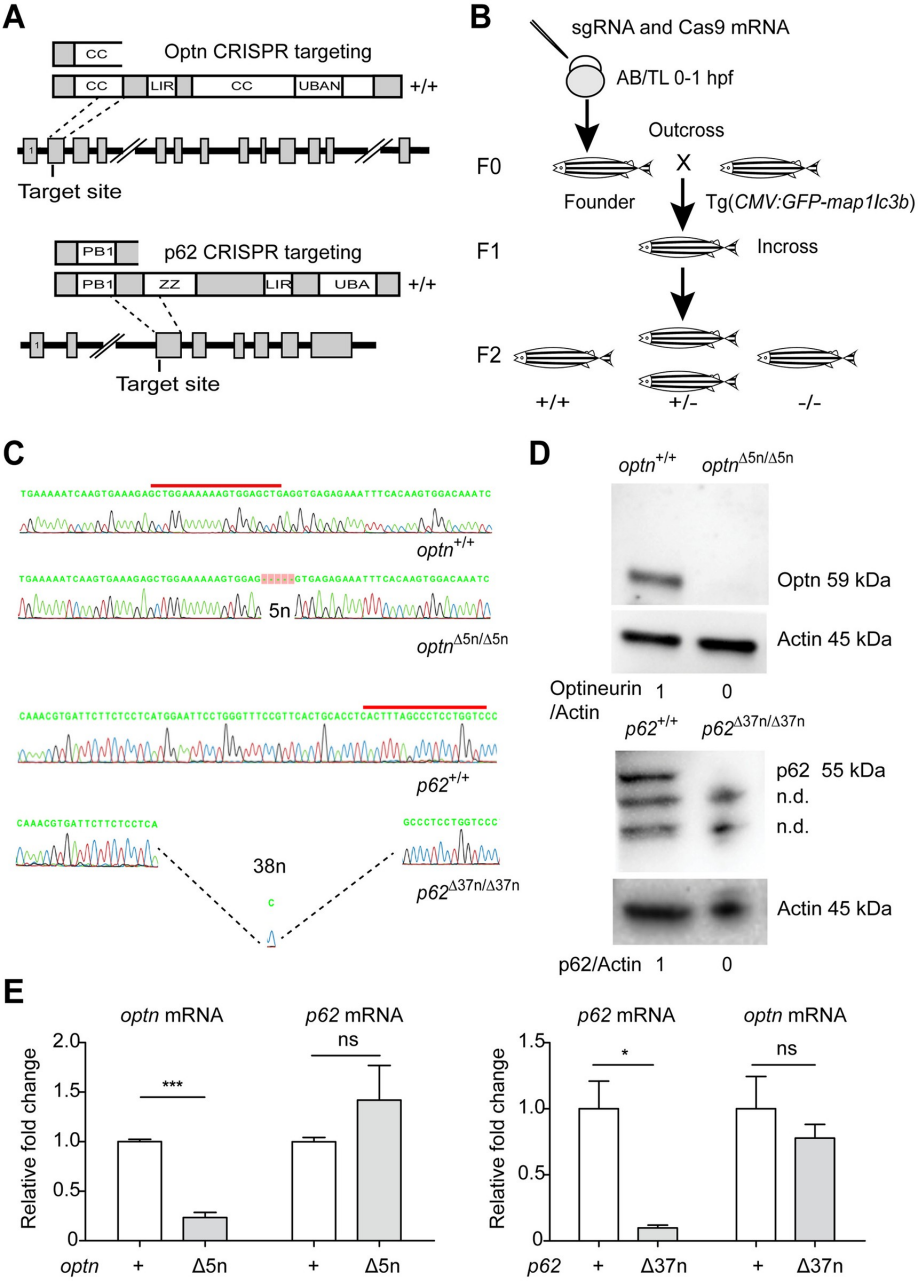
Generation of Optn and p62 mutant lines. (A) Schematic representation of the Optn and p62 genetic and protein domain architecture and CRISPR target site. Optn (517 aa) and p62 (452 aa) both contain an Lc3 interaction region domain (LIR) and ubiquitin binding domains (UBAN in Optn and UBA in p62). Additionally, two coiled-coil motifs (CC) in Optn and the PHOX/Bem1p (PB) and Zinc Finger (ZZ) domains of p62 are indicated. The gene loci are shown with coding exons as grey boxes (14 in Optn and 8 in p62) and introns as solid black lines (large introns not drawn to scale). The position of the CRISPR target site sequences at the beginning of exon 2 in *optn* and exon 3 in *p62* are indicated and the predicted truncated proteins in the mutant lines are drawn above. (B) Schematic diagram of the generation of *optn* and *p62* mutant lines. Target-specific sgRNA and Cas9 mRNAs were co-injected into one cell stage WT embryos. Founders were outcrossed to Tg(*CMV*:*GFP-map1lc3b*) fish and the F1 was incrossed to obtain homozygous mutant and wild type F2 siblings. (C) Sanger sequencing of WT and mutant F2 fish. Red lines indicate CRISPR target sites. The Optn and p62 mutant sequences contain deletions (indels) of 5 and 37 nucleotides, respectively. (D) Confirmation of CRISPR mutation effect by Western blot analysis. Protein samples were extracted from 4 dpf *optn* or 3 dpf *p62* mutant and WT larvae (>10 embryos/sample) and Western blots were repeated at least three times with independent extracts. The blots were probed with antibodies against Optn or P62 and Actin as a loading control. Optn/Actin and P62/Actin ratios) are indicated below. kDa, kilodalton. n.d., non-determined protein bands. (E) *optn* and *p62* mRNA was detected by quantitative PCR. Total RNA was isolated from 4 dpf of *optn*^+/+^, *optn*^Δ5n/Δ5n^, *p62*^+/+^ and *p62*^Δ37n/Δ37n^ embryos (>10 embryos/sample) from three biological replicates.

Since ubiquitinated bacteria are targets for members of the sequestosome-like receptor family, we compared the protein sequences of its members p62, Optn, Calcoco2 (Ndp52), Nbrc1, and Tax1bp1 between human, zebrafish and other vertebrates, showing a high overall degree of conservation (S1A Fig). We focused this study on two members of the family, *p62* and *optn*, which are transcriptionally induced during Mm infection of zebrafish based on published RNA sequencing data [27] and show strong similarity with their human orthologues in the ubiquitin-binding domains (UBA in p62 and UBAN in Optn) and Lc3 interaction regions (LIR) (S1B Fig). With the aim to investigate the functions of Optn and p62 in anti-mycobacterial autophagy, we utilized CRISPR/Cas9 genome editing technology to generate mutant zebrafish lines. We designed short guide RNAs for target sites at the beginning of coding exons 2 of the *optn* and *p62* genes, upstream of the exons encoding the ubiquitin and Lc3 binding regions, such that the predicted effect of CRISPR mutation is a complete loss of protein function (Fig 2A). A mixture of sgRNA and Cas9 mRNA was injected into zebrafish embryos at the one cell stage and founders carrying the desired mutations were outcrossed to GFP-Lc3 zebrafish (Fig 2B). The established *optn* mutant allele carried a 5 nucleotides deletion at the target site, which we named *optn*^Δ5n/Δ5n^ (ZFIN allele name *optn*^*ibl51*^) (Fig 2C). The *p62* mutant allele carried an indel resulting in the net loss of 37 nucleotides, which we named *p62*^Δ37n/Δ37n^ (ZFIN allele name *p62*^*ibl52*^) (Fig 2C). The homozygous mutants were fertile and produced embryos that did not exhibit detectable morphological differences compared with embryos produced by their wild-type (*optn*^+/+^ or *p62*^+/+^) siblings (S1C Fig). Furthermore, no significant deviation from the Mendelian 1:2:1 ratio for +/+, +/- and -/- genotypes was observed when the offspring of heterozygous incrosses were sequenced at 3 months of age (Fig 1D). Western blot analysis using anti-OPTN and anti-p62 C-terminal antibodies confirmed the absence of the proteins in the respective mutant lines (Fig 2D). In addition, quantitative PCR (Q-PCR) analysis revealed approximately 4.5-fold reduction of *optn* mRNA in the *optn*^Δ5n/Δ5n^ larvae and 10-fold reduction of *p62* mRNA in the *p62*^Δ37n/Δ37n^ larvae, indicative of nonsense-mediated mRNA decay (Fig 2E). Collectively, the *optn*^Δ5n/Δ5n^ and *p62*^Δ37n/Δ37n^ mutant zebrafish produce no functional Optn or p62, respectively, and the loss of these ubiquitin receptors does not induce detectable developmental defects that could interfere with the use of the mutant lines in infection models.

### Optn or p62 deficiencies affect autophagy

**Fig 3:**
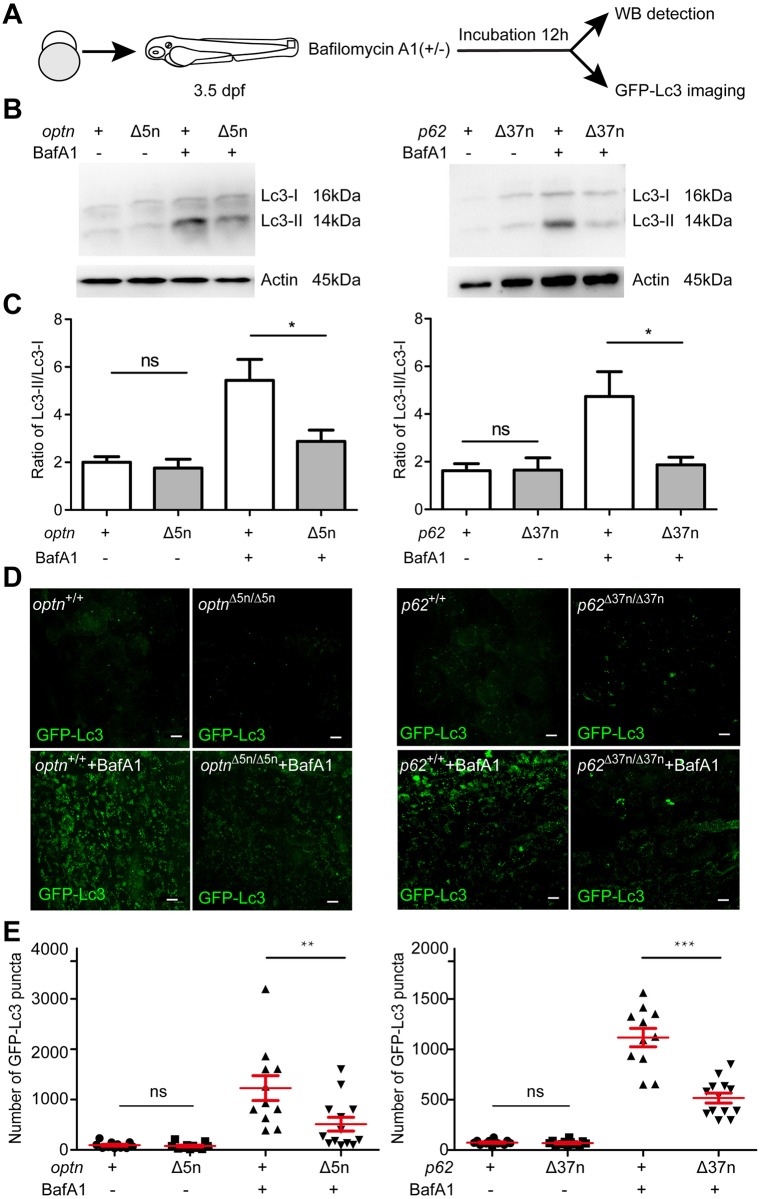
Optn or p62 deficiency affects autophagosome formation. (A) Workflow of the experiments shown in (B-G). Larvae were treated with 100 nM of Baf A1 for 12 h from 3.5 dpf. The GPF-Lc3 negative larvae were selected to assay autophagy activity by Western blot, the GFP-Lc3 positive larvae were collected to monitor autophagic activity using confocal imaging. The red square indicates the region for confocal imaging. (B) Level of basal autophagy in WT and mutant embryos in absence or presence of Baf A1. Protein samples were extracted from 4 dpf WT and mutant larvae (>10 embryos/sample). The blots were probed with antibodies against Lc3 and Actin as a loading control. Western blots were repeated at least three times with independent extracts. (C) Quantification of Lc3-II fold changes in WT and mutant embryos in absence or presence of Baf A1. Western blot band intensities were quantified by Lab Image. Data is combined from three independent experiments. (D) Representative confocal micrographs of GFP-Lc3 puncta present in the tail fin of *optn*^+/+^, *optn*^Δ5n/Δ5n^, *p62*^+/+^ and *p62*^Δ37n/Δ37n^ at 4 dpf. Scale bars, 10 μm. (E). Quantification of the number of GFP-Lc3 puncta in *optn*^+/+^, *optn*^Δ5n/Δ5n^, *p62*^+/+^ and *p62*^Δ37n/Δ37n^ larvae with and without Baf A1 treatment. Each larva was imaged at a pre-defined region of the tail fin (as indicated by the red boxed area in Fig3 A) (≥11 larvae/group). Results are accumulated from two independent experiments. ns, non-significant, *p<0.05, **p<0.01, ***p<0.001.

To analyze the effects of Optn or p62 deficiency on autophagy, we performed Lc3 Western blot detection on whole embryo extracts and imaged GFP-Lc3 signal *in vivo* (Fig 3A). Differences in the levels of the cytosolic (Lc3-I) and membrane-bound (Lc3-II) forms of Lc3 or effects on GFP-Lc3 puncta accumulation can be due to altered autophagosome formation or differences in autophagosome degradation. To distinguish between these possibilities, we examined Lc3-II/Lc3-I levels and GFP-Lc3 accumulation in the absence or presence of Bafilomycin A1 (Baf A1), which is an inhibitor of vacuolar H+ ATPase (V-ATPase) that blocks autophagosome degradation by inhibiting the fusion with lysosomes [35, 36]. First, we performed a dose range assay to determine the effect of Baf A1 on Lc3-II accumulation in zebrafish embryos. Results showed that after 12 h of incubation, a dosage of 100nM resulted in Lc3-II accumulation without affecting the Lc3-I level (S2A Fig). The accumulation of Lc3-II in the presence of Baf A1 indicates that autophagic flux is occurring in the zebrafish embryos. Because a higher dosage of Baf A1 additionally increased the Lc3-I level (S2A Fig), we utilized a dosage of 100nM to test Lc3-II accumulation in WT and mutant embryos not carrying the GFP-Lc3 reporter (Fig 3B). No differences in Lc3-II accumulation were observed between *optn*^+/+^ and *optn*^Δ5n/Δ5n^ embryos or between *p62*^+/+^ and *p62*^Δ37n/Δ37n^ embryos in the absence of Baf A1 (Fig 3C). However, Baf A1 treatment induced significantly higher accumulation of Lc3-II in WT embryos than in *optn* or *p62* mutant embryos (Fig 3C). In agreement, Baf A1 treatment resulted in accumulation of GFP-Lc3 puncta in *optn* or *p62* mutants, but a significantly higher GFP-Lc3 accumulation was detected in the corresponding WT controls (Fig 3D and 3E).

The function of Optn and p62 as ubiquitin receptors implies that these proteins are degraded themselves during the process of autophagy. Therefore, we asked if p62 protein levels are affected in *optn* mutants or, vice versa, if *p62* mutation impacts Optn protein levels. Western blot analysis showed accumulation of p62 and Optn protein in wildtype embryos in response to Baf A1 treatment, confirming that these ubiquitin receptors are substrates for autophagy under basal conditions (S2B Fig). Levels of p62 protein were reduced in *optn*^Δ5n/Δ5n^ embryos compared with *optn*^+/+^, both in absence or presence of Baf A1 (S2B Fig). This difference was not due to a transcriptional effect, since p62 mRNA levels were not significantly different between *optn*^+/+^ and *optn*^Δ5n/Δ5n^ embryos (Fig 2E). Similarly, levels of Optn protein were reduced in *p62*^Δ37n/Δ37n^ embryos compared with *p62*^+/+^ in absence or presence of Baf A1 (S2B Fig), and again this was not associated with a difference in mRNA expression (Fig 2E). In conclusion, the absence of either of the ubiquitin receptors, Optn or p62, leads to reduced protein levels of the other ubiquitin receptor, which cannot be restored by blocking lysosomal degradation. Furthermore, loss of either of the receptors leads to lower levels of Lc3-II and GFP-Lc3 accumulation when lysosomal degradation is blocked, suggesting reduced activity of the autophagy pathway in the *optn* and *p62* mutants.

### Optn or p62 deficiencies increase the susceptibility of zebrafish embryos to Mm infection

**Fig 4:**
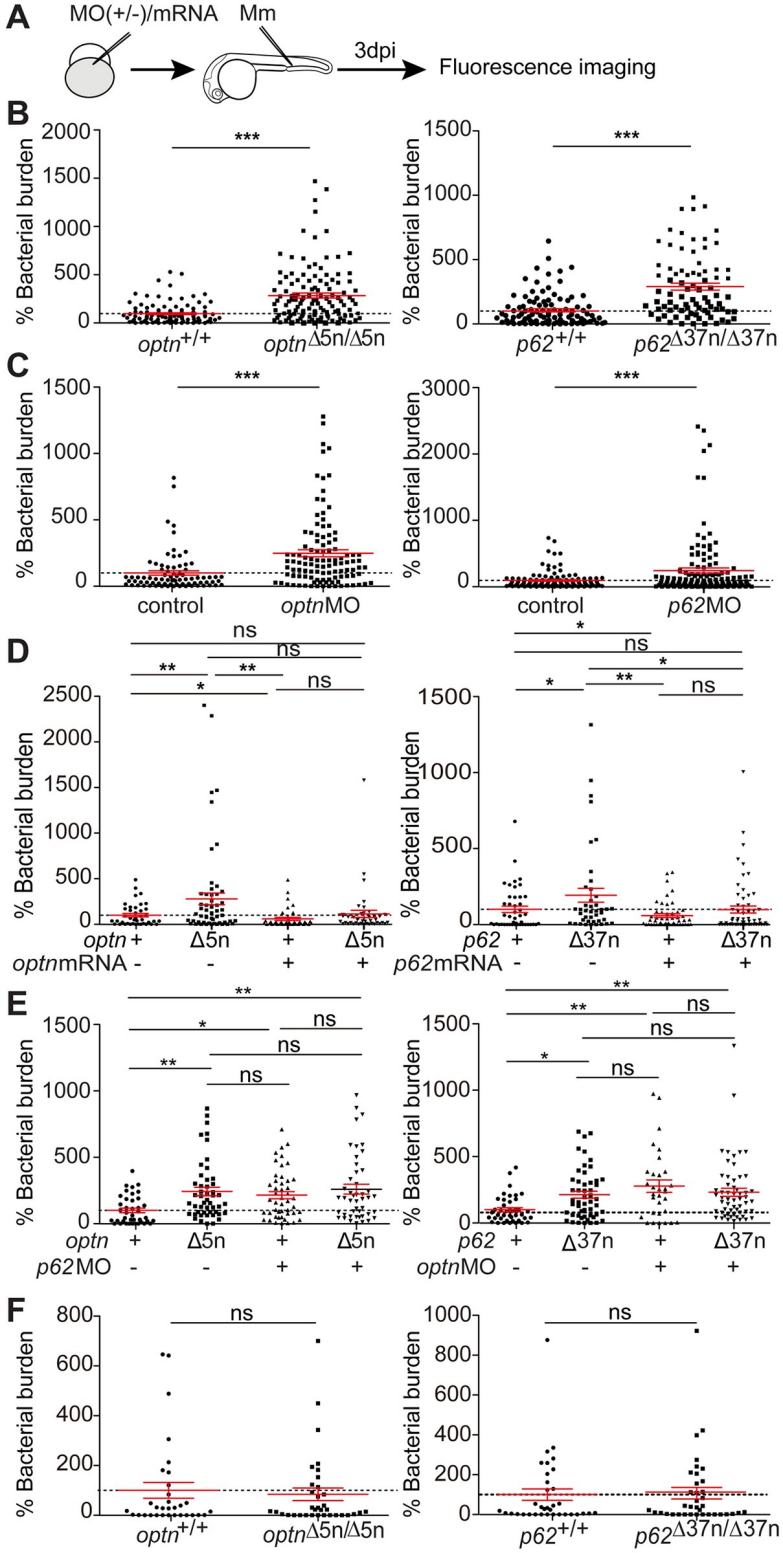
Optn or p62 deficiency leads to increased susceptibility to Mm infection. (A) Workflow of the experiments shown in (B-F). Knockdown or overexpression of *optn* or *p62* was achieved by MO or mRNA injection into the one cell stage of embryos and infection was performed at 28 hpf with 200 CFU of WT Mm or 400 CFU of Mm ΔRD1 mutant. Bacterial burden quantification was done at 3dpi. (B-E) Mm infection burden in *optn* and *p62* mutant larvae (B), under *optn* and *p62* MO knockdown conditions (C), following rescue of the mutant phenotypes by *optn* or *p62* mRNA injection (D), and following injection of *p62* MO or *optn* MO in *optn* and *p62* mutants, respectively (E). (F) Infection burden of *optn* and *p62* mutant larvae following Mm ΔRD1 injection. All data are accumulated from three experimental repeats. Each dot represents an individual larva. ns, non-significant, *p<0.05, **p<0.01, ***p<0.001.

Next, we asked if *optn* or *p62* mutations would affect the resistance of zebrafish embryos to mycobacterial infection. We injected Mm into embryos via the caudal vein at 28 hpf to measure infection burden at 3 dpi (Fig 4A). The infection data showed that *optn* or *p62* mutant embryos were hypersusceptible to Mm infection compared with their WT controls, culminating in an increase of the Mm fluorescent signal of 2.8 and 2.9 times, respectively (Fig 4B). Western blot analysis was performed to determine if mutation of *p62* or *optn* affected the protein levels of the other SLR during infection. The level of p62 was approximately 60% lower in *optn* mutants and a similar reduction was observed for Optn in *p62* mutants, indicating that reduced levels of the other SLR may contribute to the hypersusceptibility phenotype of the mutant lines (S2C Fig). In addition, we examined whether transient knockdown of *optn* or *p62* would phenocopy the infection phenotype of the mutant lines. We injected *optn* or *p62* antisense morpholino (MO) oligonucleotides into the one cell stage of embryos and collected injected individuals at 1–3 dpi for confirmation of the knockdown effect by reverse transcription polymerase chain reaction (RT-PCR) and Western blot (S3A–S3E Fig). Despite that strong reduction of mRNA and protein levels was observed only until 1 dpi, the analysis of Mm infection burden at 3 dpi showed that transient knockdown of *optn* or *p62* led to similar increases of the Mm infection burden as had been observed in the mutant lines (Fig 4C). These results suggests that differences in the resistance to Mm are already determined early in the infection process.

As a further control for the specificity of the *optn* and *p62* mutant phenotypes, we demonstrated that injection of the corresponding mRNA rescued the hypersusceptibility of the mutants to Mm infection (Fig 4D). Since OPTN and p62 are known to function cooperatively in xenophagy of *Salmonella enterica* [22–24], we next asked if double deficiency of Optn and p62 in zebrafish resulted in an increased infection burden compared to single mutation of either *optn* or *p62*. No additive effect on the infection burden was observed when *p62* MO was injected into *optn* mutant embryos or *optn* MO into *p62* mutant embryos (Fig 4E). Because Mm depends on the RD1 virulence locus to escape from phagosomes [37, 38], infection with ΔRD1 mutant bacteria is not expected to trigger selective autophagy. In agreement, infection with ΔRD1 Mm led to similar infection burdens in *optn* and *p62* mutants as in their wild type siblings (Fig 4F). Taken together, our data demonstrate that both Optn and p62 are required for controlling Mm infection and that loss of either of these ubiquitin receptors cannot be compensated for by the other receptor in this context. Furthermore, these data show that Optn and p62 specifically target Mm bacteria that have a functional RD1 virulence locus mediating phagosomal escape.

### Optn or p62 deficiency reduces the autophagy response to Mm infection

**Fig 5:**
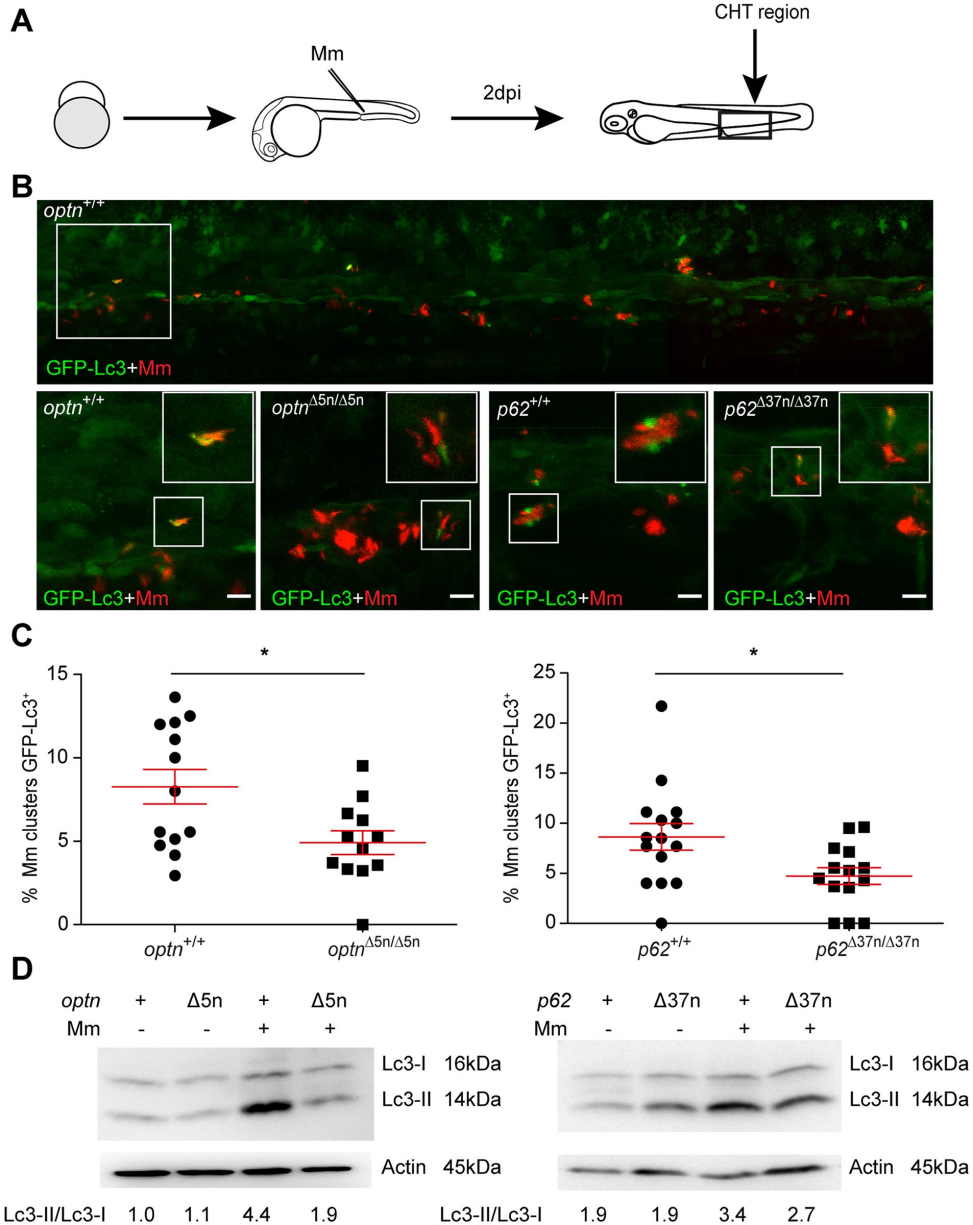
Optn or p62 deficiency inhibits targeting of Mm by GFP-Lc3. (A) Workflow of the experiment shown in B. 2 dpi fixed larvae were used for confocal imaging. The entire CHT region was imaged, as indicated by the black box. (B) Representative confocal micrographs of GFP-Lc3 co-localization with Mm clusters in infected larvae. The top image shows an overview of the CHT region in *optn*^+/+^ infected larvae. The area indicated by the white box is detailed below. The bottom images show GFP-Lc3 co-localization of Mm clusters in *optn*^+/+^, *optn*^Δ5n/Δ5n^, *p62*^+/+^ and *p62*^Δ37n/Δ37n^ infected larvae. The arrowheads indicate the overlap between GFP-Lc3 and Mm clusters. Scale bars, 10 μm. (C) Quantification of the percentage of Mm clusters positive for GFP-Lc3 vesicles. The data is accumulated from two independent experiments; each dot represents an individual larva (≥12 larvae/group). ns, non-significant, *p<0.05, **p<0.01, ***p<0.001. (D) Western blot analysis of Lc3 protein levels in infected and uninfected larvae. Protein samples were extracted from 4 dpf larvae (>10 larvae/sample). The blots were probed with antibodies against Lc3 and Actin as a loading control and Lc3-II/Lc3-I ratios are indicated below. Western blots were repeated twice with independent extracts.

Having established that mutation of either *optn* or *p62* results in increased Mm infection burden, we investigated if the inability of mutant embryos to control infection might be associated with altered inflammatory responses or is due to a reduction in the targeting of mycobacteria to autophagy. Therefore, we first examined the expression levels of 4 key markers of inflammation, *tnfa*, *il1b*, *cxcl11aa*, and *cxcl8a* (*il8*), which were selected based on previous expression profiling of Mm infection [27]. The analysis showed that the increased infection burden of *p62* and *optn* mutants was not associated with differences in the expression of these inflammatory genes (S4 Fig). Next, we investigated the autophagic targeting of mycobacteria in the mutants by examining the association of GFP-Lc3 with Mm at 1 dpi. Mm has formed small infection foci at this time point, which could be manually scored as positive or negative for GFP-Lc3 association. In wildtype (WT) embryos 5–6% of these infection foci were positive for GFP-Lc3 (S5A and S5B Fig). The percentage of GFP-Lc3 positive Mm clusters was approximately 50% lower in the *optn* or *p62* mutant embryos compared with their WT controls, but differences were not statistically significant due to the relatively low number of these GFP-Lc3 association events (S5A and S5B Fig). We continued to examine GFP-Lc3 targeting to Mm at 2 dpi and found that mutation of *optn* or *p62* resulted in significantly decreased GFP-Lc3 co-localization with Mm clusters (Fig 5A, 5B and 5C). In addition, we used GFP-Lc3-negative mutant and WT larvae for Western blot analysis of Lc3-II protein levels in response to infection. We found that Mm infection increased Lc3-II protein levels approximately 3- to 5-fold in WT (*optn*^+/+^ and *p62*^+/+^) larvae at 3 dpi, whereas this induction level was approximately 50% lower in the *optn* and *p62* mutant larvae (Fig 5D). Mm-infected mutant embryos also showed reduced Lc3-II accumulation in the presence of Baf A1 (S5C Fig). Despite the reduced GFP-Lc3 targeting, we could still detect Lysotracker-positive Mm clusters in the *optn* and *p62* mutant larvae, indicating that the phago-lysosomal pathway is not defective in these mutants (S5D Fig). Taken together, these data support the hypothesis that Optn and p62 are required for autophagic defense against mycobacterial infection.

### Overexpression of *optn* or *p62* increases resistance of zebrafish embryos to Mm infection

**Fig 6:**
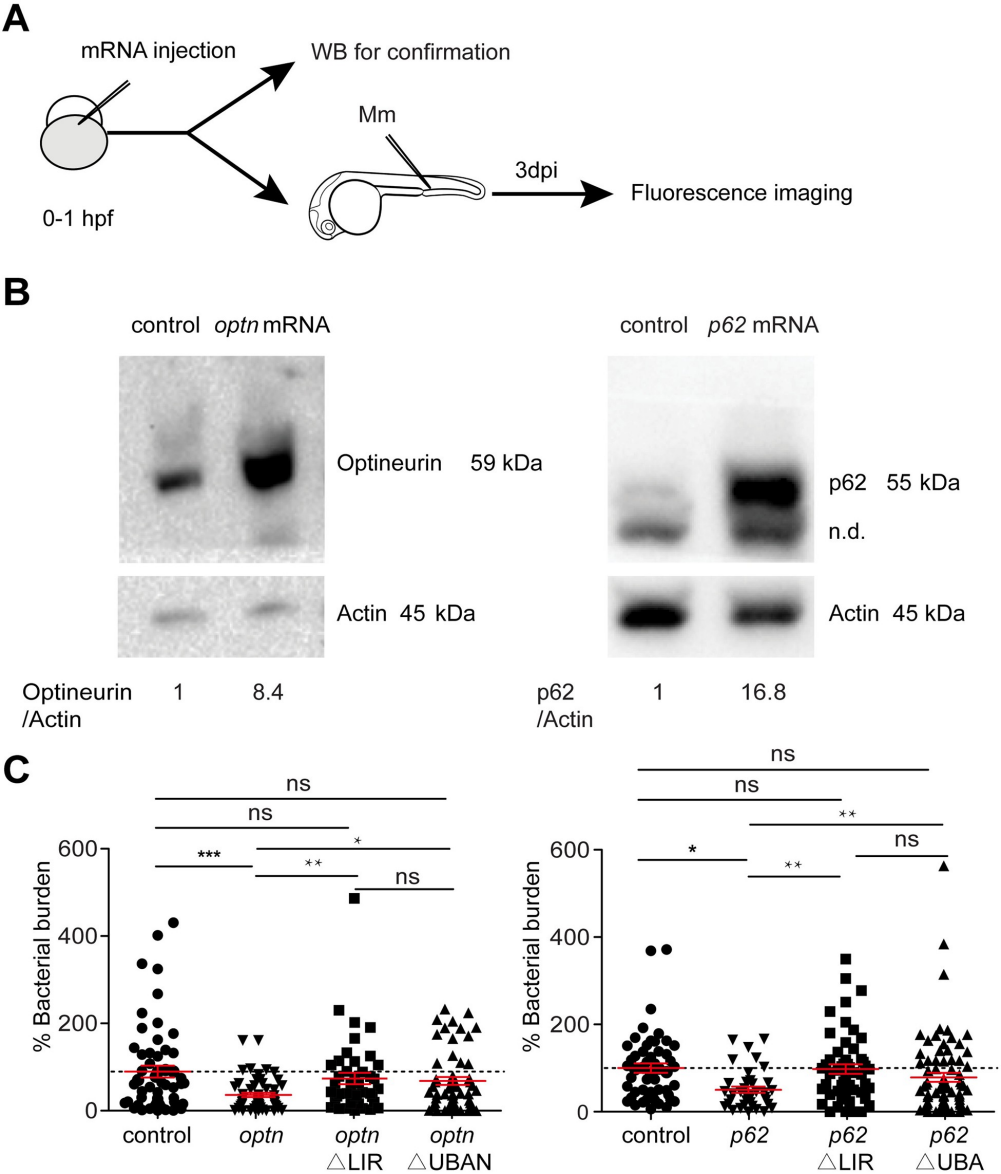
Transient overexpression *optn* or *p62* mRNA protects against Mm infection. (A) Workflow representing the experimental design in (B-C). *optn* or *p62* mRNA was injected into the one cell stage of WT embryos. Injected embryos were collected at 28 hpf for confirmation of the overexpression by Western blot analysis. Embryos were infected at 28 hpf with 200 CFU Mm and bacterial burden was determined at 3 dpi. (B) Western blot analysis to test the effect of transient overexpression of *optn* or *p62* mRNA. Protein extracts were made from >20 mRNA-injected or control embryos per group. The blots were probed with antibodies against Optn or p62 and Actin as a loading control. Similar results were observed in two independent experiments. n.d., non-determined protein bands. (C) Quantification of Mm infection burden in embryos injected with full length or ΔLIR/ΔUBA(N) deletion mRNAs of *optn* and *p62*. Accumulated data from three independent infection experiments is shown. ns, non-significant, *p<0.05, **P<0.01, ***p<0.001.

To further test the hypothesis that Optn and p62 mediate autophagic defense against Mm, we generated full-length *optn* and *p62* mRNAs *in vitro* and injected these into embryos at the one cell stage, resulting in ubiquitous overexpression (Fig 6A). The increase in Optn or p62 protein levels following mRNA injection was verified by Western blot analysis (Fig 6B). Overexpression of *optn* or *p62* mRNAs significantly reduced Mm infection burden at 3 dpi compared to the control groups (Fig 6C). Furthermore, injection of *optn* or *p62* mRNAs carrying deletions in the sequences encoding the ubiquitin binding domains or Lc3 interaction regions did not lead to a reduction of the Mm infection burden compared with the control groups (Fig 6C and S6A Fig). Thus, we conclude that *optn* or *p62* overexpression protects against Mm infection in a manner dependent on the interaction of the Optn and p62 proteins with both ubiquitin and Lc3.

### Overexpression of *optn* or *p62* promotes GFP-Lc3 association with Mm

**Fig 7:**
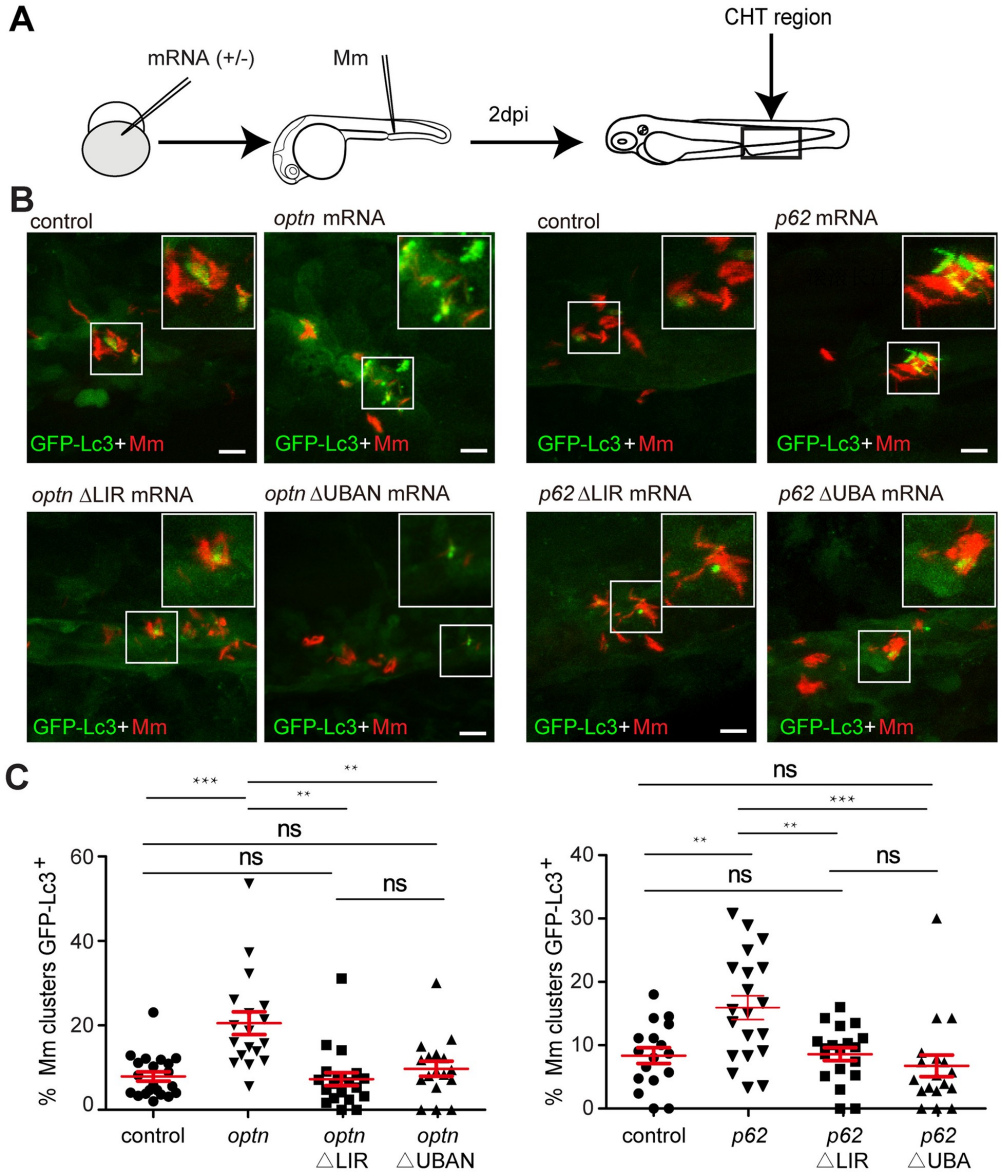
Transient overexpression of *optn* or *p62* mRNA promotes GFP-Lc3 recruitment to Mm clusters. (A) Workflow of the experiments in (B-C). *optn* or *p62* mRNA was injected into the one cell stage of embryos and 2 dpi fixed larvae were used for confocal imaging. The entire CHT region was imaged, as indicated by the black box. (B) Representative confocal micrographs of GFP-Lc3 co-localization with Mm clusters in larvae injected with full length or ΔLIR/ΔUBA(N) deletion mRNAs of *optn* and *p62*. The arrowheads indicate the overlap between GFP-Lc3 and Mm clusters. Scale bars, 10 μm. (C) Quantification of the percentage of Mm clusters positive for GFP-Lc3 vesicles. Each dot represents an individual larva (≥18 larvae/group). Data are accumulated from two independent experiments. ns, non-significant, *p<0.05, **P<0.01, *** p<0.001.

Since overexpression of *optn* or *p62* mRNAs resulted in decreased Mm infection burden, we postulated that elevation of the Optn or p62 protein levels would result in increased targeting of Mm to autophagy by these ubiquitin receptors, in a manner dependent on the functions of the Lc3 interaction (LIR) and ubiquitin binding domains (UBAN/UBA). To test this hypothesis, we injected the full-length mRNAs, or mRNAs generated from deletion constructs lacking these domains, and quantified GFP-Lc3-positive and GFP-negative Mm infection foci at 1 dpi and 2 dpi (Fig 7A–7C, S6B and S6C Fig). The results showed that overexpression of full-length *optn* or *p62* mRNAs significantly increased the percentage of GFP-Lc3-positive Mm clusters at 2 dpi, compared with the control groups (Fig 7B and 7C). Conversely, injection of *optn* ΔUBAN, *optn* ΔLIR, *p62* ΔUBA and *p62* ΔLIR mRNAs did not increase the association of GFP-Lc3 with Mm clusters (Fig 7B and 7C). Similar results could be observed as early as 1 dpi (S6B and S6C Fig). In conclusion, our combined results demonstrate that Optn and p62 can target Lc3 to Mm and that increasing the level of either of these receptors promotes host defense against this mycobacterial pathogen.

## Discussion

Members of the family of sequestosome (p62/SQSTM1)-like receptors (SLRs) function in autophagic host defense mechanisms targeting a range of intracellular pathogens, including *Salmonella*, *Shigella*, *Streptococci*, *Listeria*, *Mycobacteria*, and Sindbis virus [5, 13, 14, 39]. These discoveries inspired investigations into autophagy modulators as host-directed therapeutics for treatment of infectious diseases, including Tb [9, 40, 41]. However, the relevance of autophagic defense mechanisms for host resistance against Mtb infection has recently been questioned [15, 42]. This indicates that there are significant gaps in our understanding of the interaction between components of the autophagy pathway and mycobacterial pathogens, emphasizing the need for more research in animal models of Tb [12]. Here, we have studied the function of two SLR family members in the zebrafish Mm infection model. We show that selective autophagy mediated by p62 and Optn provides resistance against mycobacterial infection in the context of our *in vivo* infection model that is representative of the early stages of Tb granuloma formation [17, 19]. Our findings support the host-protective role of p62 in Tb by autophagic targeting of *Mycobacteria*, in line with previous *in vitro* studies [13, 14]. Importantly, we also present the first evidence linking Optn to resistance against *Mycobacteria*, expanding our understanding of the function of SLRs in host defense against intracellular pathogens.

The zebrafish embryo and larval Tb model provides the opportunity to image critical stages of the mycobacterial infection process, from the initial phagocytosis of Mm by macrophages up to the early stages of Tb granuloma formation [43]. The model is representative of miliary Tb, where the infection is disseminated to multiple organs of the host. The embryonic and larval stages of the zebrafish allow us to study the contribution of innate immunity to host defense, since they lack a matured adaptive immune response at this time point of development [17]. We therefore used this model to study the importance of autophagic defense mechanisms during innate host defense against mycobacterial infections. In this study, we successfully generated *p62* and *optn* loss-of-function zebrafish mutant lines using CRISPR/Cas9 technology. Besides its role in host defense, p62 is a stress-inducible protein that functions as a signalling hub in diverse processes like amino acid sensing and the oxidative stress response [44]. Defects in autophagy pathways caused by mutations in *OPTN* have been associated with human disorders like glaucoma, Paget disease of bone, and amyotrophic lateral sclerosis [24, 45]. Despite the important functions reported for p62 and OPTN in cellular homeostasis, the mutant fish lines we generated are viable and fertile. The absence of either p62 or Optn resulted in reduced protein levels of the other ubiquitin receptor, even when lysosomal degradation was blocked. The mechanism responsible for this cross-talk currently remains unknown. In addition, loss of either of the receptors leads to lower levels of Lc3-II and GFP-Lc3 accumulation when lysosomal degradation is blocked, indicating that mutation of either *p62* or *optn* is sufficient to reduce activity of the autophagy pathway in zebrafish larvae. Therefore, we could use these mutant lines to gain a better understanding of the role of p62, Optn, and selective autophagy in host defense against mycobacterial infection.

Genetic links between autophagy pathway genes and susceptibility to Tb in human populations support the function of autophagy in innate host defense against Mtb [46]. However, the contribution of autophagy as a direct anti-mycobacterial mechanism has recently been challenged, since macrophage-specific depletion of a number of autophagy genes, including *p62*, did not affect the outcome of disease in a mouse model of Tb [15, 42]. A possible explanation for these findings, as suggested by the authors of this study, is that Mtb, like other successful intracellular pathogens, could have evolved virulence mechanisms that subvert or exploit autophagic defense mechanisms employed by the host [47]. In case of one of the autophagy genes, *ATG5*, macrophage-specific depletion increased Mtb infection in mice by over-activating inflammation rather than by impairing autophagic processes [15]. It is therefore conceivable that modulating the activity of SLRs could also affect inflammation. Indeed, OPTN has been implicated in inflammatory bowel disease and both p62 and OPTN are involved in regulation of inflammatory signaling downstream of NF-κB [48–52]. Through a process that involves polyubiquitination of regulatory proteins, both p62 and OPTN can modulate the activity of the IKK kinase complex that activates NFκB [48, 49]. We therefore investigated the expression of several inflammatory cytokine and chemokine genes that are targets of NFκB signaling. The upregulation of these genes during Mm infection was unaffected by *optn* and *p62* mutation. Nevertheless, we cannot rule out the possibility that more subtly altered inflammatory responses in *p62* and *optn* mutants could explain (part of) the increase in mycobacterial burden observed in zebrafish hosts, while the beneficial role for autophagic defense mechanisms targeting the bacteria might be limited.

To investigate the possible role of Optn and p62 in anti-mycobacterial autophagy, we quantified the association between GFP-Lc3 and Mm under loss-of-function and gain-of-function conditions of both receptors. During Mm infection in zebrafish, GFP-Lc3 puncta are frequently detected in close vicinity of intracellular bacteria, but only a small percentage of Mm bacteria are found inside vesicles with autophagic morphology as determined by electron microscopy [20, 21]. In agreement, only 3–5% of the bacteria co-localized with autophagic vesicles one day after a systemic infection of WT zebrafish embryos with mycobacteria. Although the number of GFP-Lc3 positive bacterial clusters rises over the next two days, the percentage of bacteria targeted by autophagy at any distinct time point remained relatively low (e.g. ~10% at 2 dpi). According to these results, the host only employs autophagic defense mechanisms against a small proportion of the invading mycobacteria during early stages of the infection, either because there is no greater need, or because the pathogens are indeed effectively suppressing this response. It is important to note though that GFP-Lc3 association with Mm is a transient process [20], which means that the percentage of bacteria that encounter autophagic defenses throughout the early infection process might be much higher. Strikingly, the percentage of bacteria labeled by ubiquitin closely resembled the percentage of bacteria targeted by autophagy, and we were able to detect clear colocalization between ubiquitin and GFP-Lc3 at bacterial clusters. Upon loss-of-function of either p62 or Optn, the co-localization between bacteria and autophagic vesicles decreased and the bacterial burden increased. Conversely, overexpression of either ubiquitin binding receptor increased autophagic targeting of bacteria and resulted in lower bacterial burdens, both of which required the presence of functional Lc3 and ubiquitin binding domains. Taken together, we conclude that autophagic targeting of Mm by p62 and Optn indeed provides protection against infection in the zebrafish model.

Mm and Mtb share the RD1 virulence locus, which encodes the ESX1 secretion system that is required for bacterial translocation from phagosomes into the cytosol [37, 38, 53] The autophagy machinery can directly entrap cytosolic bacteria that become ubiquitinated after phagosomal escape, or it can delay the escape process by membrane repair of permeabilized and ubiquitinated phagosomes [54]. In agreement, we found that *optn* and *p62* mutants and their WT siblings were equally susceptible to infection with the escape incompetent RD1 mutant strain. While our results implicate SLRs in the autophagic defense against cytosolic Mm, another mechanism for targeting these bacteria to degradative vesicles has also been reported. The RD1-dependent phagosomal escape of Mm in bone-marrow derived macrophages triggered bacterial ubiquitination and subsequent targeting to LAMP1-positive vesicles in an ATG5-independent manner [38]. Besides this autophagy-independent sequestration, Mm bacteria were also observed inside classical double-membraned autophagosomes, suggesting that SLR-mediated targeting also occurs in this model. It is possible that autophagy-dependent and -independent routes of targeting cytosolic Mm also occur in zebrafish and that actin polymerization might protect a subset of bacteria against sequestration, as has been found in the study of macrophages [38]. We observed acidification of part of the Mm-containing vesicles in both WT and SLR mutant zebrafish, but the experimental tools are currently lacking to further investigate the fate of Mm after sequestration in autophagic or other compartments. Because mycobacteria are known for being able to largely tolerate the acidic environment that results from lysosome fusion [35], it is possible that the major contribution of autophagy to bacterial killing might not be made by xenophagic delivery of Mm to acidic lysosomes, but by SLR-mediated delivery of ubiquitinated peptides that have strong anti-microbial activity against mycobacteria [7].

In summary, our findings on Mm infection in zebrafish confirm that p62 mediates ubiquitin-dependent autophagic targeting of mycobacteria in an *in vivo* model for early stages of Tb granuloma formation. We also provide the first evidence that the SLR family member Optn is involved in autophagic targeting of ubiquitinated mycobacteria. The effects of *optn* and *p62* mutation cannot be entirely separated, since we found that mutation of one SLR reduced protein levels of the other. Nevertheless, our data suggest that Optn and p62 have non-redundant and possibly cooperative functions in defense against Mm. This is consistent with the distinct functions of these SLRs in the selective autophagy response to *Salmonella* Typhimurium and *Listeria monocytogenes* [22, 23, 55]. Furthermore, a direct interaction between the two SLRs has been demonstrated in a study showing that tumor-suppressor HACE1, a ubiquitin ligase, ubiquitinates OPTN and promotes its interaction with p62 to form the autophagy receptor complex [56]. While expression of major inflammatory markers was unaffected by p62 or Optn deficiency, we have shown that the autophagic targeting of Mm by these ubiquitin-binding receptors forms an important aspect of innate host defense against Tb in the zebrafish model. Our results are therefore especially important for the development of new treatment strategies for Tb patients with a compromised adaptive immune system–such as in HIV-coinfection. Based on these results, selective autophagy stimulation remains a promising strategy for development of novel anti-Tb therapeutics.

## Materials and methods

### Ethics statement

Zebrafish lines in this study (S1 Table) were handled in compliance with local animal welfare regulations as overseen by the Animal Welfare Body of Leiden University (License number: 10612) and maintained according to standard protocols (zfin.org). All protocols adhered to the international guidelines specified by the EU Animal Protection Directive 2010/63/EU. The generation of zebrafish *optn*^*ibl51*^ and *p62*^*ibl52*^ mutant lines was approved by the Animal Experimention Committee of Leiden University (UDEC) under protocol 14198. All experiments with these zebrafish lines were done on embryos or larvae up to 5 days post fertilization, which have not yet reached the free-feeding stage. Embryos were grown at 28.5°C and kept under anesthesia with egg water containing 0.02% buffered 3-aminobenzoic acid ethyl ester (Tricaine, Sigma) during bacterial injections, imaging and fixation.

### CRISPR/Cas9 mediated mutagenesis of zebrafish *optn* and *p62*

Single guide RNAs (sgRNAs) targeting the second coding exon of zebrafish *optn* (ENSDART00000014036.10) and the third coding exon of *p62* (ENSDART00000140061.2) were designed using the chop-chop website [57]. To make sgRNAs, the template single strand DNA (ssDNA) (122 bases) was obtained by PCR complementation and amplification of full length ssDNA oligonucleotides. Oligonucleotides up to 81 nucleotides were purchased from Sigma-Aldrich using standard synthesis procedures (25 nmol concentration, purification with desalting method) (S2 and S3 Tables). The pairs of semi-complimentary oligos were annealed together by a short PCR program (50 μL reaction, 200uM dTNPs, 1 unit of Dream Taq polymerase (EP0703, ThermoFisher); PCR program: initial denaturation 95°C/3 minute (min), 5 amplification cycles 95°C/30 Second (s), 55°C/60 s, 72°C/30 s, final extension step 72°C/15 min) and subsequently the products were amplified using the primers in S2 Table with a standard PCR program (initial denaturation 95°C/3 min, 35 amplification cycles 95°C/30 s, 55°C/60 s, 72°C/30 s, final extension step 72°C/15 min). The final PCR products were purified with Quick gel extraction and PCR purification combo kit (00505495, ThermoFisher). The purified PCR products were confirmed by gel electrophoresis and Sanger sequencing (Base Clear, Netherlands). For *in vitro* transcription of sgRNAs, 0.2 μg template DNA was used to generate sgRNAs using the MEGA short script ^®^T7 kit (AM1354, ThermoFisher) and purified by RNeasy Mini Elute Clean up kit (74204, QIAGEN Benelux B.V., Venlo, Netherlands). The Cas9 mRNA was transcribed using mMACHINE^®^ SP6 Transcription Kit (AM1340, Thermo Fisher) from a Cas9 plasmid (39312, Addgene) (Hrucha et al 2013) and purified with RNeasy Mini Elute Clean up kit (74204, QIAGEN Benelux B.V., Venlo, Netherlands). A mixture of sgRNA and Cas9 mRNA was injected into one cell stage WT embryos (sgRNA 150 pg/embryo and Cas9 mRNA 300 pg/embryo). The effect of CRISPR injection was confirmed by PCR and Sanger sequencing.

### Genomic DNA isolation and genotyping

Genomic DNA was isolated from an individual embryo (2 dpf) or small pieces of the tail fin tissue of adults (>3 months) by fin clipping. Embryos or tissue samples were incubated in 200 μL 100% Methanol at -20°C overnight (O/N), then methanol was removed, and remaining methanol was evaporated at 70°C for 20 min. Next, samples were incubated in 25 μL of TE buffer containing 1.7 μg/μL proteinase K at 55°C for more than 5 h. Proteinase K was heat inactivated at 80°C for 30 min, after which samples were diluted with 100 μL of Milli-Q water. Genotyping was performed by PCR-amplification of the region of interest using the primers in S5 Table followed by Sanger sequencing to identify mutations (Base Clear, Netherlands).

### Western blot analysis

Embryos or larvae were anaesthetised with Tricaine (Lot#MKBG4400V, SIGMA-ALDRICH) and homogenised with a Bullet-blender (Next-Advance) in RIPA buffer (#9806, Cell Signalling) containing a protein inhibitor cocktail (000000011836153001, cOmplete, Roche). The extracts were then spun down at 4°C for 10 min at 12000 rpm/min and the supernatants were frozen for storage at -80°C. Western blot was performed using Mini-PROTEAN-TGX (456–9036, Bio-Rad) or 18% Tris-Hcl 18% polyacrylamide gels, and protein transfer to commercial PVDF membranes (Trans-Blot Turbo-Transfer pack, 1704156, Bio-Rad). Membranes were blocked with 5% dry milk (ELK, Campina) in Tris buffered saline (TBS) solution with Tween 20 (TBST, 1XTBS contains 0.1% Tween 20) buffer and incubated with primary and secondary antibodies. Digital images were acquired using Bio-Rad Universal Hood II imaging system (720BR/01565 UAS). Band intensities were quantified by densitometric analysis using Image Lab Software (Bio-Rad, USA) and values were normalised to actin as a loading control. Antibodies used were as follows: polyclonal rabbit anti-OPTN (C-terminal) (1:200, lot#100000; Cayman Chemical), polyclonal rabbit anti-p62 (C-terminal) (PM045, lot#019, MBL), polyclonal rabbit anti Lc3 (1:1000, NB100-2331, lot#AB-3, Novus Biologicals), Anti mono-and polyubiquitinated conjugates mouse monoclonal antibody (1:200; BML-PW8810-0100, lot#01031445, Enzo life Sciences), Polyclonal actin antibody (1:1000, 4968S, lot#3, Cell Signaling), Anti-rabbit IgG, HRP-Linked Antibody (1:1000, 7074S, Lot#0026, Cell Signaling), Anti-mouse IgG, HRP-linked Antibody (1:3000, 7076S, Lot#029, Cell Signaling).

### MO design and validation

*optn* and *p62* splice blocking MOs were purchased from Gene Tools. For MO sequences see S4 Table. MOs were diluted in Milli Q water with 0.05% phenol red and 0.1 pmol *optn* or 0.5 pmol *p62* MO was injected in a volume of 1 nL into the one cell stage of embryos as previously described [21]. The knockdown effect was validated by RT-PCR and Western blot.

### Infection conditions and bacterial burden quantification

Infections of zebrafish embryos were performed by microinjection into the blood island at 28 hpf as previously described [58], using the mCherry-labeled Mm 20 strain, the mCrimson-labeled Mm M strain, and the mCherry labeled Mm ΔRD1 strain [59]. The injection dose was 200 CFU for experiments with WT Mm bacteria and 400 CFU for infections with the ΔRD1 mutant. Before the injection, embryos were manually dechorionated around 24 hpf. Approximately 5 min before bacterial injections, zebrafish embryos were brought under anaesthesia with tricaine. Infected embryos were imaged using a Leica MZ16FA stereo fluorescence microscopy with DFC420C camera, total fluorescent bacterial pixels per infected fish were determined on whole-embryo stereo fluorescent micrographs using previously described software [60].

### Confocal laser scanning microscopy and image quantification

Fixed or live embryos were mounted with 1.5% low melting agarose (140727, SERVA) and imaged using a Leica TCS SPE confocal microscope. For quantification of basal autophagy, fixed uninfected 4 dpf larvae were imaged by confocal microscopy with a 63x water immersion objective (NA 1.2) in a pre-defined region of the tail fin to detect GFP-LC3-positive vesicles (Fig 3D and 3E). The number of GFP-Lc3 vesicles per condition was quantified using Fiji/ImageJ software (Fig 3D and 3E). For quantification of the autophagic response targeted to Mm clusters (Fig 1B and 1C, S4A, S4B, S6A and S6B Figs), live or fixed infected embryos were viewed by confocal microscopy with a 63x water immersion objective (NA 1.2) and the number of Mm clusters that were targeted by GFP-Lc3 puncta in the tail region were counted manually. The same approach was used to quantify Ubiquitin targeting to Mm clusters (Fig 1E and 1F). To quantify the percentage of GFP-Lc3^+^ Mm clusters, we imaged the entire caudal hematopoietic tissue (CHT) region of 2 dpi larvae (confocal microscopy; 40X water immersion objective with NA 1.0) and stitched multiple images together to manually count the number of Mm clusters positive for GFP-Lc3 out of the total number of clusters (Figs 5B, 5C, 7B and 7C).

### Immunostaining

Embryos (1,2,3 dpi) were fixed with 4% PFA in PBS and incubated overnight with shaking at 4ᵒC. After washing the embryos three times briefly in PBS with 0.8% Triton-x100) (PBSTx), the embryos/larvae were digested in 10 μg/ml proteinase K (000000003115879001, SIGMA-ALDRICH) for 10 minutes at 37ᵒC. Subsequently, the embryos were quickly washed, blocked with PBSTx containing 1% Bovine serum albumins (BSA) (A4503-100g, SIGMA-ALDRICH) for 2h at room temperature and incubated overnight at 4ᵒC in mono-and polyubiquitinated conjugates mouse monoclonal antibody (1:200; BML-PW8810-0100; Enzo lifes Siences), diluted in the blocking buffer. Next, embryos were washed three times in PBSTx, incubated for 1 h in blocking buffer at room temperature, incubated for 2 h at room temperature in 1:200 dilution of Alexa Fluor 488 or 633 goat anti-mouse (Invitrogen) in blocking buffer, followed with three times washes in PBSTx for imaging.

### mRNA preparation and injection

*optn* (ENSDART00000014036.10, Ensembl) and *p62* (ENSDART00000140061.2, Ensembl) cDNAs were amplified from 3 dpf WT embryos by PCR (primers in S5 Table) and ligated into a vector using the Zero-blunt cloning PCR kit (450245, Invitrogen). The sequence was confirmed by Sanger sequencing (BaseClear, Netherlands), after which *optn* and *p62* cDNAs were subcloned into a pCS2+ expression vector. *optn* ΔUBAN cDNA was produced by in vitro transcription of *optn*-pCS2+ constructs digested by Sca1(R3122, NEB), which excludes the region encoding the UBAN protein domain. *optn* ΔLIR cDNA was amplified from *optn*-pCS2+ constructs by designed primers (S5 Table), excluding the LIR protein domain. The PCR products were gel purified by Quick gel Extraction PCR Purification Combo Kit (K220001, Invitrogen) and the two fragments and pCS2+ plasmid were digested by BamH1(R0136S, NEB) and EcoR1(R0101S, NEB), after which the two fragments were ligated into pCS2+ plasmid by T4 DNA ligase. *p62* ΔUBA cDNA was obtained from a *p62*-pCS2+ construct by Nco1(R0193S, NEB) digestion and religation, which excludes the region encoding the UBA protein domain. *p62* ΔLIR cDNA was obtained from a *p62*-pCS2+ construct by NcoN1 digestion and religation. *Optn* mRNA, *optn* ΔUBAN, and *optn* ΔLIR mRNA was generated using SP6 mMessage mMachine kit (Life Technologies) from Kpn1 or Sac1(R0156S, NEB) digested *optn*–pCS2+ constructs. RNA purification was performed using the RNeasy Mini Elute Clean up kit (QIAGEN Benelux B.V., Venlo, Netherlands). *In vitro* transcription of *p62*, *p62* ΔUBA, and *p62* ΔLIR was performed using mMESSAGE mMACHINE T3 Transcription Kit (AM1348, Thermo Fisher) and purified using the RNeasy MiniElute Cleanup kit (QIAGEN Benelux B.V., Venlo, Netherlands). All mRNAs were injected into one cell stage embryos at a dose of 100 pg in a volume of 1 nL, and the overexpression effects of *optn* or *p62* were validated by Q-PCR and Western blot.

### Gene expression analysis

Total RNA was extracted using Trizol reagent (15596026, Invitrogen) according to the manufacturer’s instructions and purified with RNeasy Min Elute Clean up kit (Lot:154015861, QIAGEN). RNAs were quantified using a NanoDrop 2000c instrument (Thermo Scientific, U.S). Reverse transcription reaction was performed using 0.5 μg of total RNA with iScript cDNA synthesis kit (Cat:#170–8891, Bio-Rad). The mRNA expression level was determined by quantitative real-time PCR using iQSYBR Green Supermix (Cat:170–8882, Rio-Rad) and Single color Real-Time PCR Detection System (Bio-Rad, U.S) as previously described [61]. All primers are listed in S5 Table.

### Statistical analyses

Statistical analyses were performed using GraphPad Prism software (Version 5.01; GraphPad). All experimental data (mean ± SEM) was analyzed using unpaired, two-tailed t-tests for comparisons between two groups and one-way ANOVA with Tukey’s multiple comparison methods as a posthoc test for comparisons between more than two groups. (ns, no significant difference; *p < 0.05; **p < 0.01; ***p < 0.001). To determine whether the offspring of F1 heterozygous mutants follows Mendelian segregation, the obtained data was analysed with a Chi-square test (ns, no significant difference).

## Supporting information

S1 Fig Optn and p62 are highly conserved between zebrafish and human. (A) Protein sequence identity of SLRs between zebrafish and human. The percentage identity and similarity was calculated using a Clustal Omega alignment. (B) Alignment of LIR, UBAN and UBA motifs from the Optn and p62 sequences of different vertebrates. Amino acid sequences of the LIR motifs of Optn and p62 from the indicated species were aligned using Mega7 software (DNASTAR, Madison, WI) with the Clustal W2 method (EMBL, Cambridge, UK). The ubiquitin binding domains of Optn or p62 were determined by NCBI-BLASTP (https://blast.ncbi.nlm.nih.gov/Blast.cgi?PAGE=ProTeins). (C) Representative images of *WT* and mutant F2 larvae at 4 dpf. Scale bars, 250 μm. (D) Segregation from F1 heterozygous incross. Genotypes of adult fish (>3 months) combined from 4 (for *optn*) or 3 (*p62*) independent breedings were confirmed by PCR and sequencing.
(TIF)

S2 Fig Characterization of *optn* and *p62* mutant lines. (A) Validation of Baf A1 effect on zebrafish by Western blot. Baf A1 treatment at dosages of 20, 100 and 400 nM was performed by incubation for 12h in egg water. The protein samples were extracted from 4 dpf WT larvae (>10 embryos/sample). The blots were probed with antibodies against Lc3 and Actin. (B) Detection of p62 or Optn protein in mutant lines in absence or presence of Baf A1. Protein samples were extracted from *optn*^+/+^, *optn*^Δ5n/Δ5n^, *p62*^+/+^ and *p62*^Δ37n/Δ37n^ larvae at 4 dpf (>10 embryos/sample). The blots were probed with antibodies against Optn, p62 and Actin as a loading control. Optn/Actin and p62/Actin ratios are indicated below. n.d., non-determined protein bands. (C) Detection of p62 or Optn protein level in mutant larvae infected with Mm. Protein samples were extracted at 3dpi (>10 larvae/sample). The blots were probed with antibodies against Optn, p62 and Actin as a loading control. Optn/Actin and p62/Actin ratios are indicated below. The results are representative of two independent experiments. n.d., non-determined protein bands.
(TIF)

S3 Fig Injection of *optn* or *p62* MO transiently knocks down the corresponding mRNA and protein. (A) Workflow representing the experimental design in (B-E). *optn* or *p62* MOs were injected into one cell stage WT embryos, and injected embryos were collected for confirmation of the knockdown effect by RT-PCR and Western blot analysis (>20 embryos /sample). (B) Validation of the effect of *optn* splice-blocking MO e2i2 (targeting the splice event between exon 2 and intron 2) by RT-PCR on (a) the WT control group, (b) embryos injected with 0.1mM MO, or (c) embryos injected with 0.15 mM MO. The WT PCR product is 400 bp in length. (C) Validation of the effect of *p62* splice-blocking MO i1 e2 (targeting the splice event between intron 1 and exon 2) by RT-PCR on (a) the WT control group, (b) embryos injected with 0.5mM MO. The WT PCR product is 200 bp in length. (D and E) Validation of MO knockdown effect by Western blot analysis. The protein samples were extracted from 1, 3 and 5 dpf WT embryos/larvae injected with *optn* or *p62* MO (>20 individuals/sample). The blots were probed with antibodies against Optn or P62 and Actin. Optn/Actin and p62/Actin ratios are indicated below. n.d., non-determined protein bands.
(TIF)

S4 Fig Deficiency of Optn or p62 does not affect the expression of major inflammatory response genes during Mm infection. (A) Inflammatory cytokines/chemokines were detected by quantitative PCR. Total RNA was isolated at 3dpi from *optn*^+/+^, *optn*^Δ5n/Δ5n^, *p62*^+/+^ and *p62*^Δ37n/Δ37n^ larvae (>10 /sample) from three biological replicates. Mutant larvae were infected either with the same dose of Mm as their WT siblings (200 CFU) or with a lower dose (150 CFU) in order to compare inflammatory gene expression both under conditions of increased bacterial burden in the mutants or under conditions of similar bacterial burden between mutants and WT. (B) Bacterial burdens of larvae infected with different doses of Mm for analysis of inflammatory gene expression. Mm infection burden was determined at 3 dpi and data is accumulated from three replicates. Each dot represents an individual larva. ns, non-significant, *p<0.05, **P<0.01, *** p<0.001.
(TIF)

S5 Fig Optn or p62 mutation reduces autophagosome formation during Mm infection. (A) Representative confocal micrographs of GFP-Lc3 co-localization with Mm clusters in *optn*^+/+^, *optn*^Δ5n/Δ5n^, *p62*^+/+^ and *p62*^Δ37n/Δ37n^ infected embryos at 1 dpi. The arrowheads indicate the overlap between GFP-Lc3 and Mm clusters. Scale bars, 10 μm. (B) Quantification of the percentage of Mm co-localizing with GFP-Lc3 in infected embryos at 1 dpi (>6 embryo/group). ns, non-significant, *p<0.05, **P<0.01, ***p<0.001. (C) Autophagy activity in Mm-infected embryos. Protein samples were obtained from 3 dpi *optn*^+/+^, *optn*^Δ5n/Δ5n^, *p62*^+/+^ and *p62*^Δ37n/Δ37n^ infected larvae with Baf A1 12 h treatment (>10 larvae/sample). The blots were probed with antibodies against Lc3 and Actin. (D) Representative confocal images of LysoTracker staining performed on Mm-infected *optn*^+/+^, *optn*^Δ5n/Δ5n^, *p62*^+/+^ and *p62*^Δ37n/Δ37n^ larvae at 3 dpi. Scale bars, 10 μm.
(TIF)

S6 Fig Transient overexpression of full length but not ΔLIR/ΔUBA(N) mRNAs of *optn* or *p62* results in increased recruitment of GFP-Lc3 to Mm clusters. (A) Validation of transient overexpression effect of full length or ΔLIR/ΔUBA(N) deletion mRNAs of *optn* and *p62* by quantitative PCR. mRNAs were injected into the one cell stage of WT embryos and samples were collected at 28 hpf (>20 embryos/sample). Data are based on two replicates. (B) Representative confocal micrographs of GFP-Lc3 co-localization with Mm clusters in mRNA-injected larvae at 1 dpi. The arrowheads indicate the overlap between GFP-Lc3 and Mm clusters. Scale bars, 10 μm. (C) Quantification of the percentage of Mm clusters positive for GFP-Lc3 vesicles. ns, non-significant, *p<0.05, **P<0.01, *** p<0.001. Data are accumulated from two independent experiments (>15embryo/group).
(TIF)

S1 Table Zebrafish lines used in this study. (DOCX)

S2 Table Target sites for CRISPR/Cas 9 systems. (DOCX)

S3 Table Primers for complementation and amplification of sgRNA. (DOCX)

S4 Table MO sequences. (DOCX)

S5 Table Primers used in this study. (DOCX)

S6 Table Accession numbers of selective autophagy receptors. (DOCX)
