# Supplementary figures and images for: The selective autophagy receptors Optineurin and p62 are both required for zebrafish host resistance to mycobacterial infection
Source: PLoS Pathog. 2019 Feb 28;15(2):e1007329. doi: 10.1371/journal.ppat.1007329 (PMC6413957; doi:10.1371/journal.ppat.1007329)

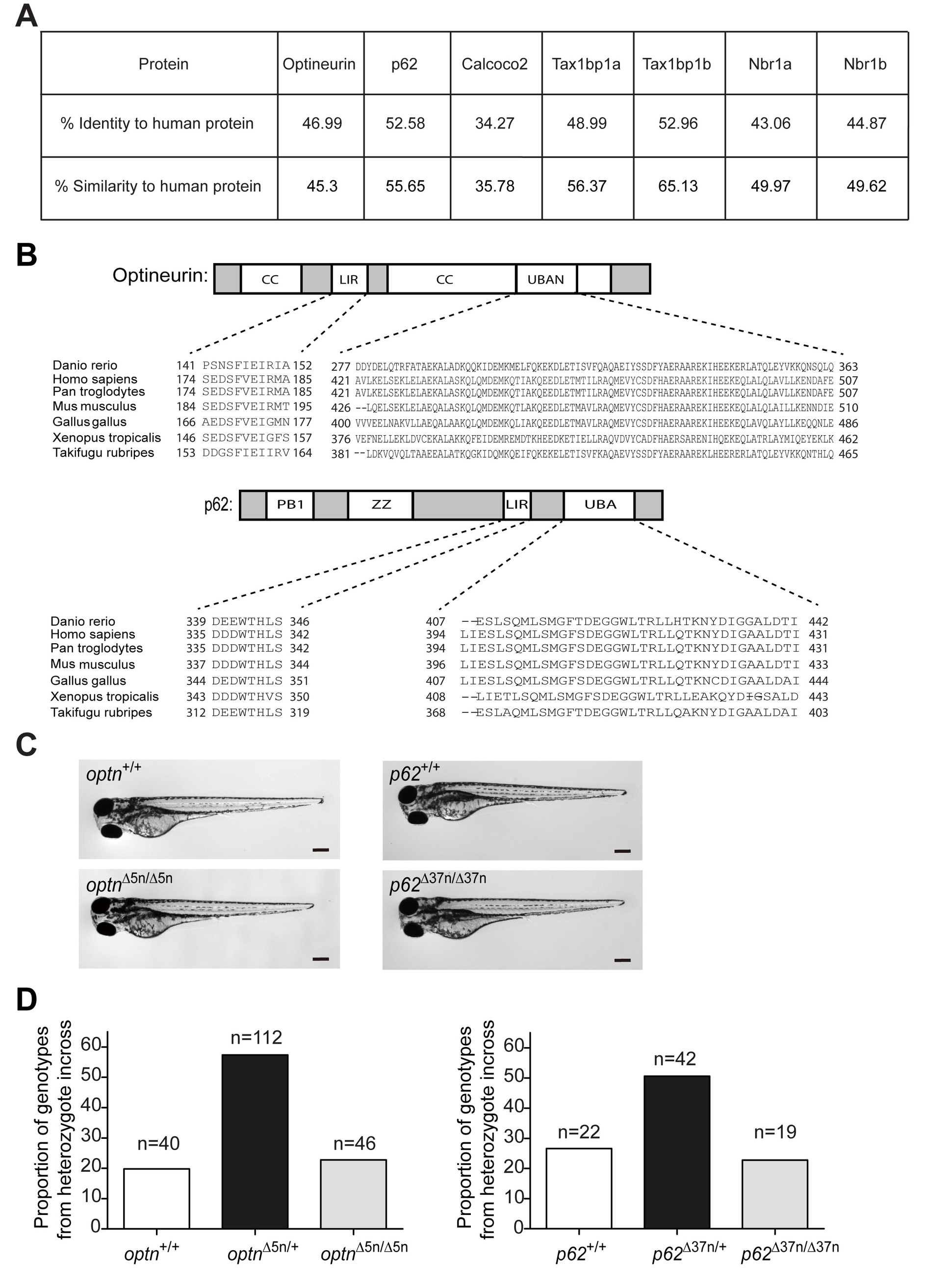

Supplement: S1 Fig — (A) Protein sequence identity of SLRs between zebrafish and human. The percentage identity and similarity was calculated using a Clustal Omega alignment. (B) Alignment of LIR, UBAN and UBA motifs from the Optn and p62 sequences of different vertebrates. Amino acid sequences of the LIR motifs of Optn and p62 from the indicated species were aligned using Mega7 software (DNASTAR, Madison, WI) with the Clustal W2 method (EMBL, Cambridge, UK). The ubiquitin binding domains of Optn or p62 were determined by NCBI-BLASTP (https://blast.ncbi.nlm.nih.gov/Blast.cgi?PAGE=ProTeins). (C) Representative images of WT and mutant F2 larvae at 4 dpf. Scale bars, 250 μm. (D) Segregation from F1 heterozygous incross. Genotypes of adult fish (>3 months) combined from 4 (for optn) or 3 (p62) independent breedings were confirmed by PCR and sequencing. (TIF) [file ppat.1007329.s001.tif]

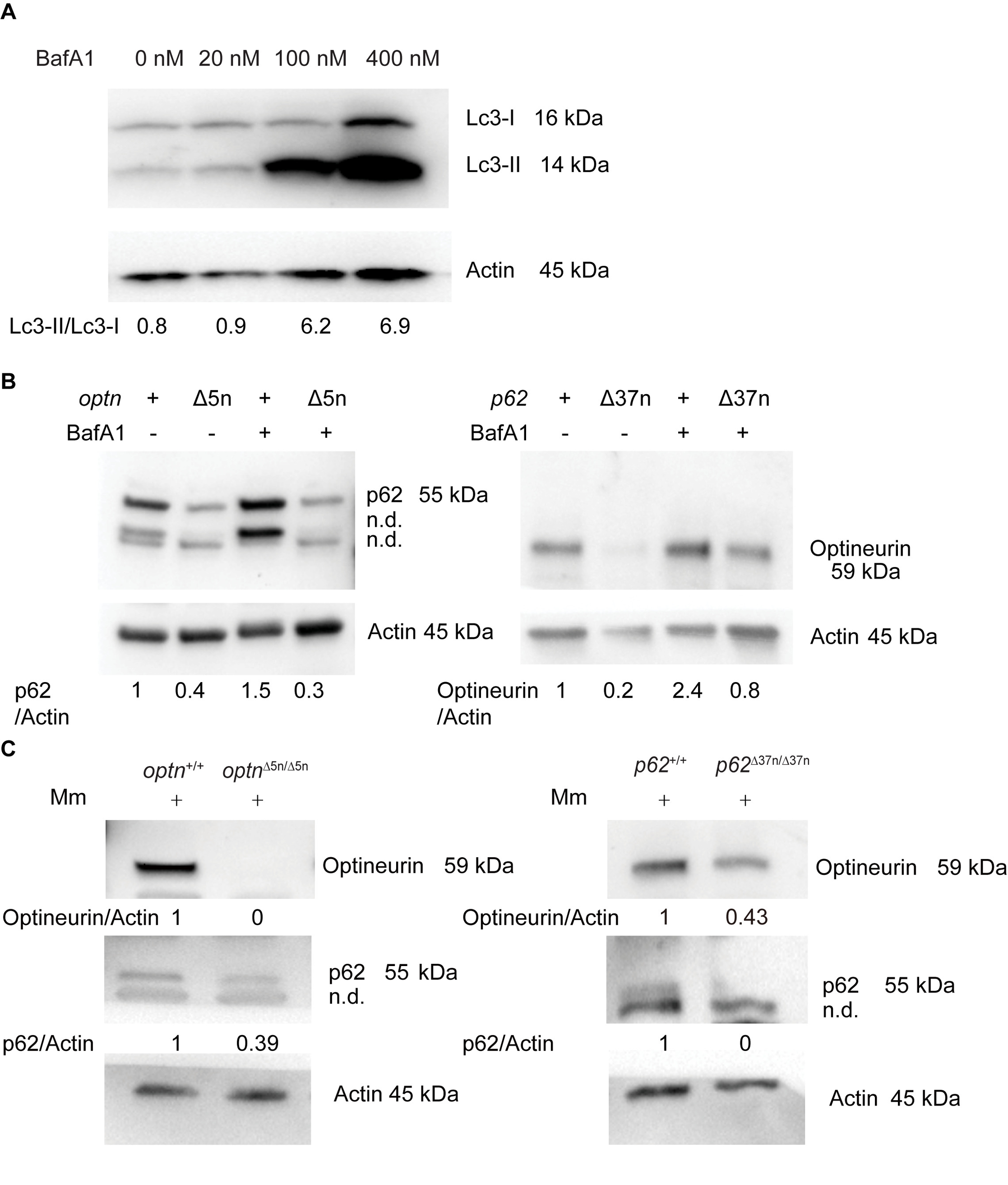

Supplement: S2 Fig — (A) Validation of Baf A1 effect on zebrafish by Western blot. Baf A1 treatment at dosages of 20, 100 and 400 nM was performed by incubation for 12h in egg water. The protein samples were extracted from 4 dpf WT larvae (>10 embryos/sample). The blots were probed with antibodies against Lc3 and Actin. (B) Detection of p62 or Optn protein in mutant lines in absence or presence of Baf A1. Protein samples were extracted from optn+/+, optnΔ5n/Δ5n, p62+/+ and p62Δ37n/Δ37n larvae at 4 dpf (>10 embryos/sample). The blots were probed with antibodies against Optn, p62 and Actin as a loading control. Optn/Actin and p62/Actin ratios are indicated below. n.d., non-determined protein bands. (C) Detection of p62 or Optn protein level in mutant larvae infected with Mm. Protein samples were extracted at 3dpi (>10 larvae/sample). The blots were probed with antibodies against Optn, p62 and Actin as a loading control. Optn/Actin and p62/Actin ratios are indicated below. The results are representative of two independent experiments. n.d., non-determined protein bands. (TIF) [file ppat.1007329.s002.tif]

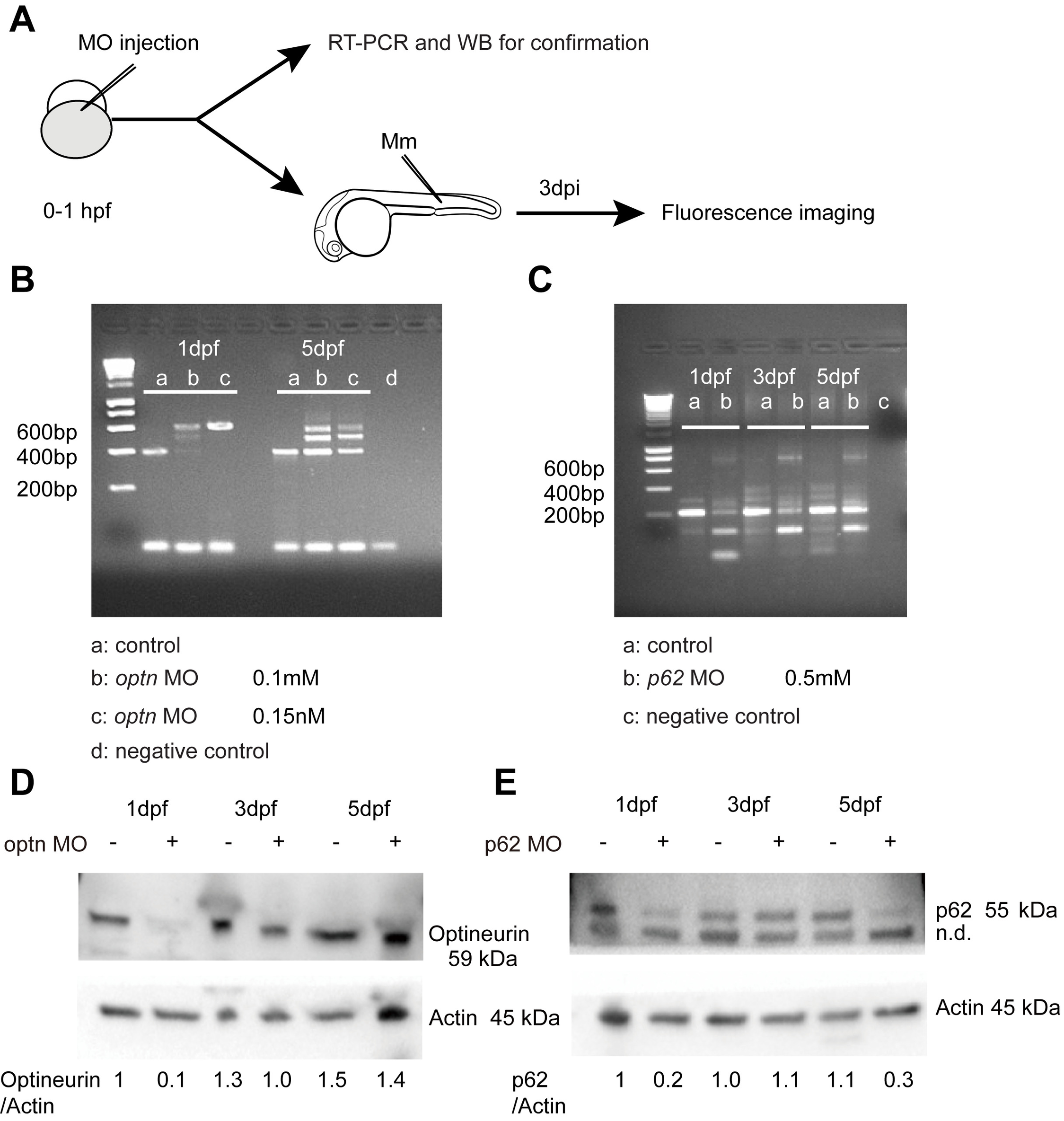

Supplement: S3 Fig — (A) Workflow representing the experimental design in (B-E). optn or p62 MOs were injected into one cell stage WT embryos, and injected embryos were collected for confirmation of the knockdown effect by RT-PCR and Western blot analysis (>20 embryos /sample). (B) Validation of the effect of optn splice-blocking MO e2i2 (targeting the splice event between exon 2 and intron 2) by RT-PCR on (a) the WT control group, (b) embryos injected with 0.1mM MO, or (c) embryos injected with 0.15 mM MO. The WT PCR product is 400 bp in length. (C) Validation of the effect of p62 splice-blocking MO i1 e2 (targeting the splice event between intron 1 and exon 2) by RT-PCR on (a) the WT control group, (b) embryos injected with 0.5mM MO. The WT PCR product is 200 bp in length. (D and E) Validation of MO knockdown effect by Western blot analysis. The protein samples were extracted from 1, 3 and 5 dpf WT embryos/larvae injected with optn or p62 MO (>20 individuals/sample). The blots were probed with antibodies against Optn or P62 and Actin. Optn/Actin and p62/Actin ratios are indicated below. n.d., non-determined protein bands. (TIF) [file ppat.1007329.s003.tif]

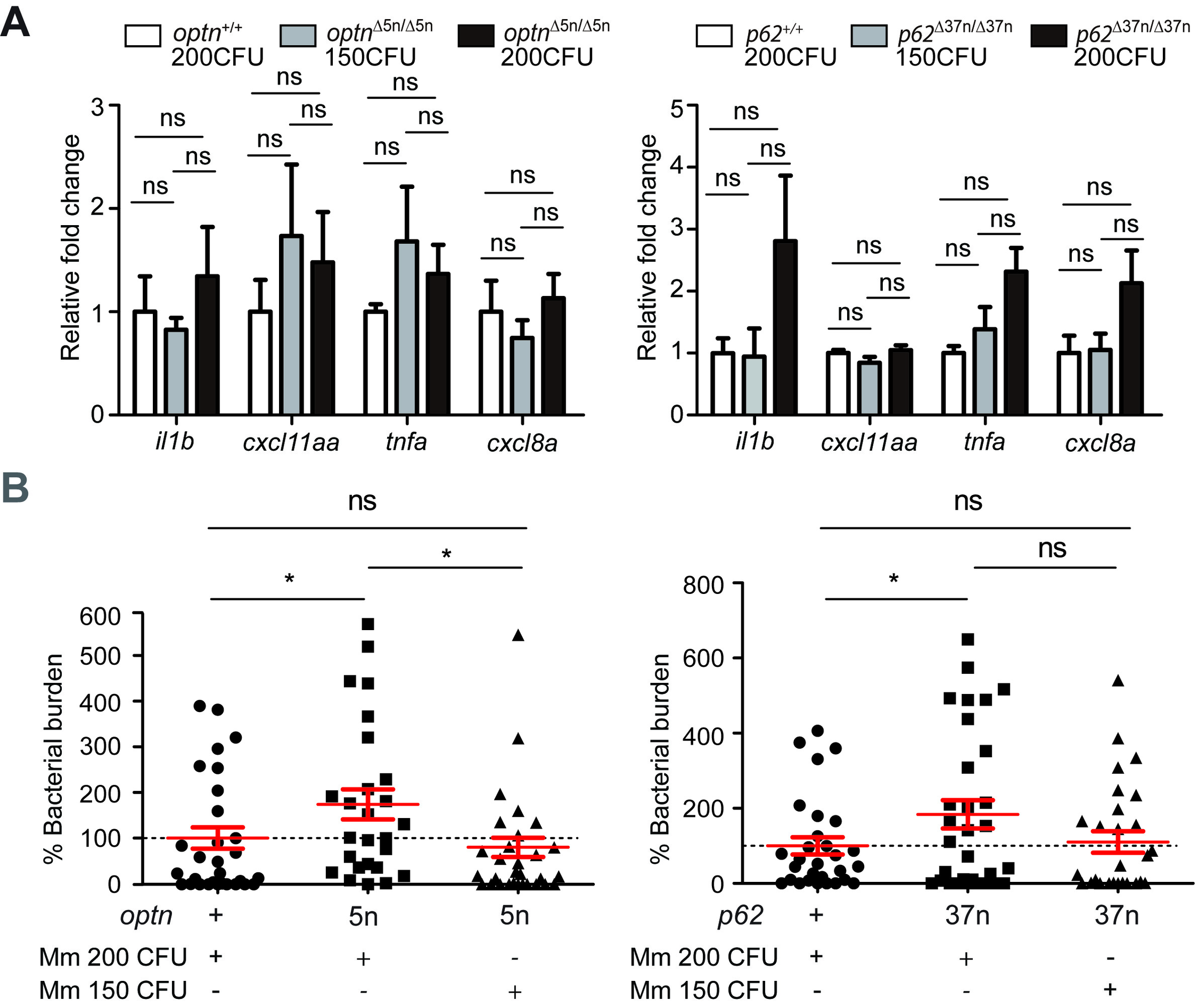

Supplement: S4 Fig — (A) Inflammatory cytokines/chemokines were detected by quantitative PCR. Total RNA was isolated at 3dpi from optn+/+, optnΔ5n/Δ5n, p62+/+ and p62Δ37n/Δ37n larvae (>10 /sample) from three biological replicates. Mutant larvae were infected either with the same dose of Mm as their WT siblings (200 CFU) or with a lower dose (150 CFU) in order to compare inflammatory gene expression both under conditions of increased bacterial burden in the mutants or under conditions of similar bacterial burden between mutants and WT. (B) Bacterial burdens of larvae infected with different doses of Mm for analysis of inflammatory gene expression. Mm infection burden was determined at 3 dpi and data is accumulated from three replicates. Each dot represents an individual larva. ns, non-significant, *p<0.05, **P<0.01, *** p<0.001. (TIF) [file ppat.1007329.s004.tif]

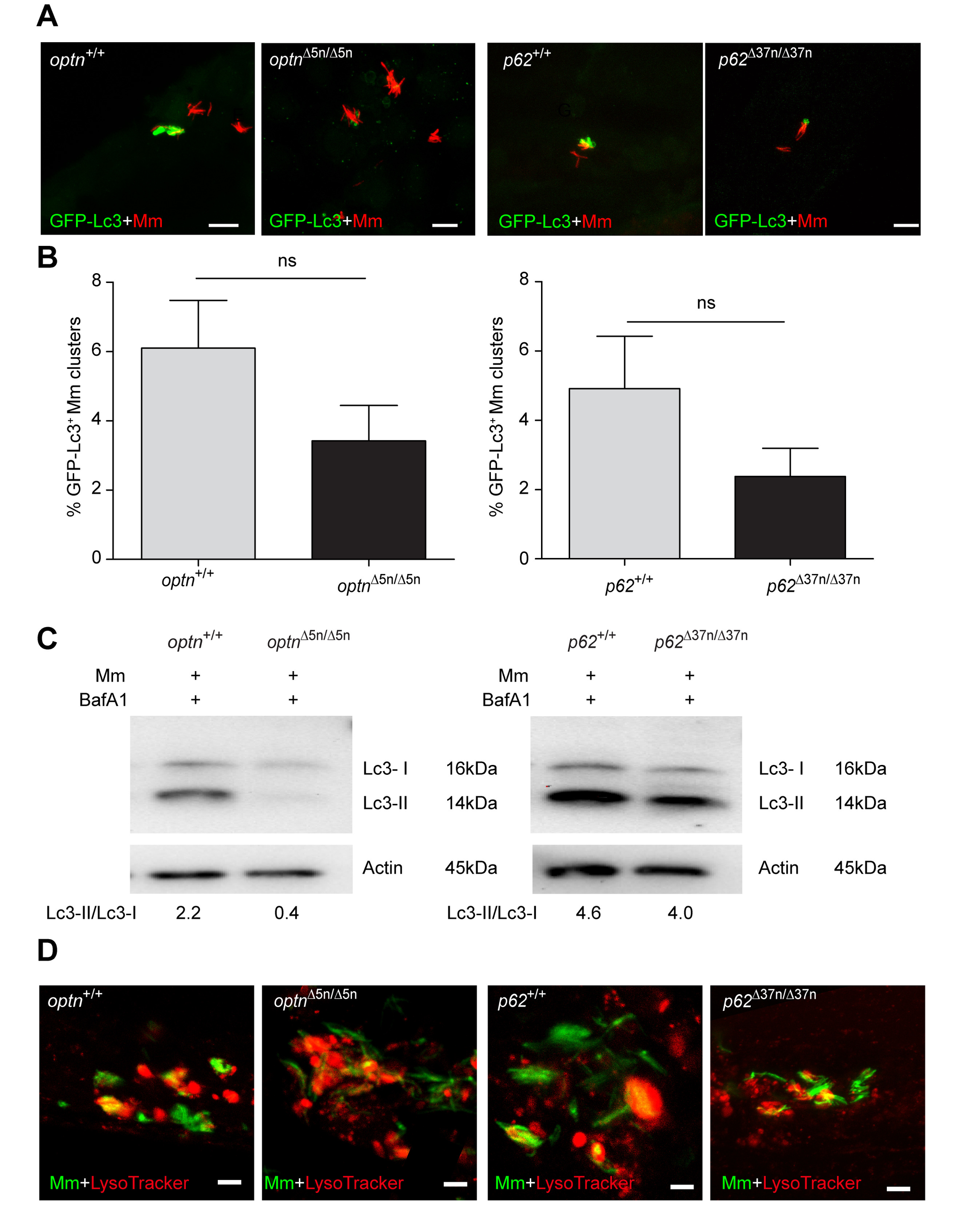

Supplement: S5 Fig — (A) Representative confocal micrographs of GFP-Lc3 co-localization with Mm clusters in optn+/+, optnΔ5n/Δ5n, p62+/+ and p62Δ37n/Δ37n infected embryos at 1 dpi. The arrowheads indicate the overlap between GFP-Lc3 and Mm clusters. Scale bars, 10 μm. (B) Quantification of the percentage of Mm co-localizing with GFP-Lc3 in infected embryos at 1 dpi (>6 embryo/group). ns, non-significant, *p<0.05, **P<0.01, ***p<0.001. (C) Autophagy activity in Mm-infected embryos. Protein samples were obtained from 3 dpi optn+/+, optnΔ5n/Δ5n, p62+/+ and p62Δ37n/Δ37n infected larvae with Baf A1 12 h treatment (>10 larvae/sample). The blots were probed with antibodies against Lc3 and Actin. (D) Representative confocal images of LysoTracker staining performed on Mm-infected optn+/+, optnΔ5n/Δ5n, p62+/+ and p62Δ37n/Δ37n larvae at 3 dpi. Scale bars, 10 μm. (TIF) [file ppat.1007329.s005.tif]

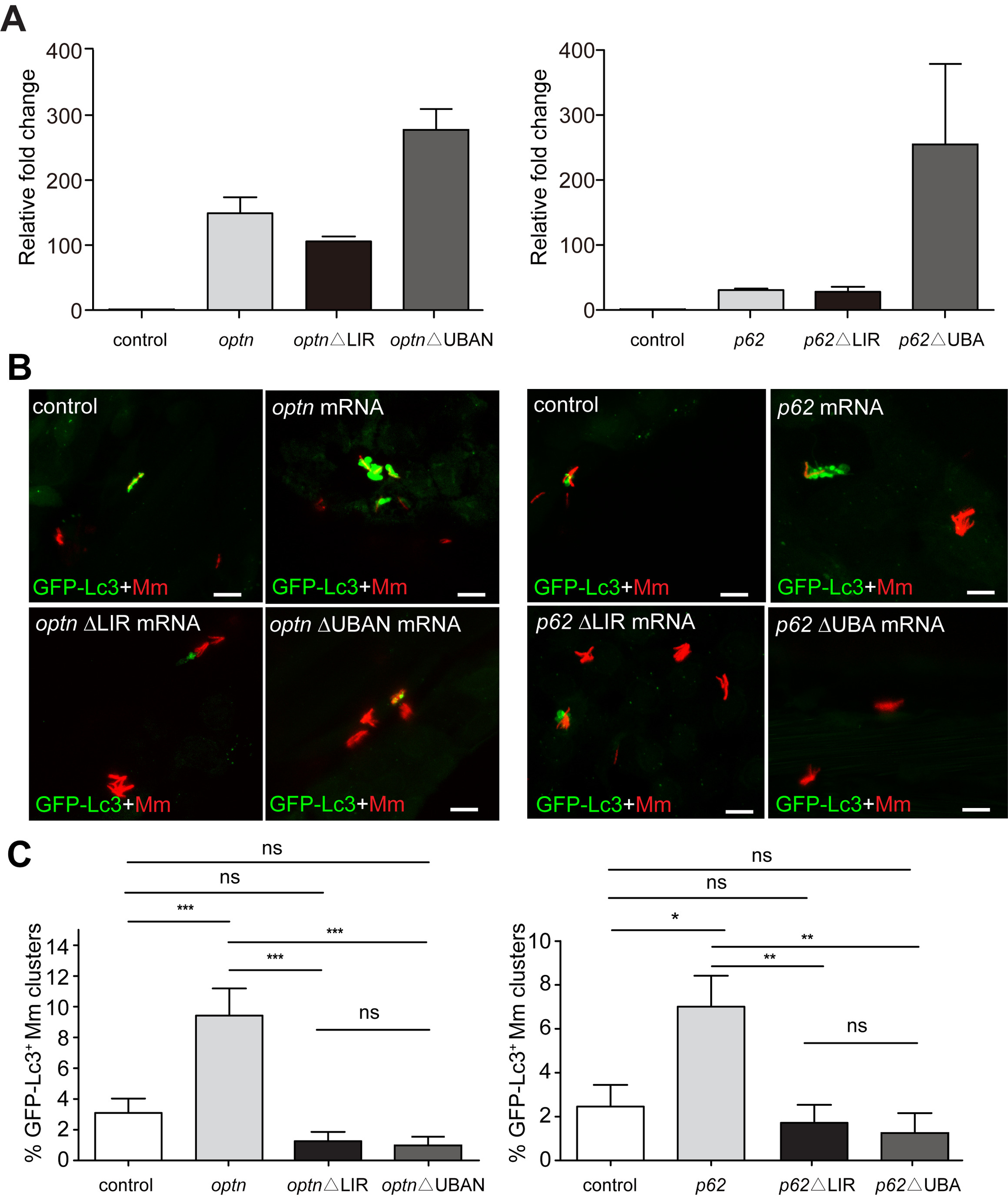

Supplement: S6 Fig — (A) Validation of transient overexpression effect of full length or ΔLIR/ΔUBA(N) deletion mRNAs of optn and p62 by quantitative PCR. mRNAs were injected into the one cell stage of WT embryos and samples were collected at 28 hpf (>20 embryos/sample). Data are based on two replicates. (B) Representative confocal micrographs of GFP-Lc3 co-localization with Mm clusters in mRNA-injected larvae at 1 dpi. The arrowheads indicate the overlap between GFP-Lc3 and Mm clusters. Scale bars, 10 μm. (C) Quantification of the percentage of Mm clusters positive for GFP-Lc3 vesicles. ns, non-significant, *p<0.05, **P<0.01, *** p<0.001. Data are accumulated from two independent experiments (>15embryo/group). (TIF) [file ppat.1007329.s006.tif]
