# Supplementary material for: The selective autophagy receptors Optineurin and p62 are both required for zebrafish host resistance to mycobacterial infection
Source: PLoS Pathog. 2019 Feb 28;15(2):e1007329. doi: 10.1371/journal.ppat.1007329 (PMC6413957; doi:10.1371/journal.ppat.1007329)
Supplement: S1 Table — (DOCX) [file ppat.1007329.s007.docx]

**S1 Table. Zebrafish lines used in this study**

| Name | Description | Reference |
| --- | --- | --- |
| WT | Wildtype strain AB/TL | 21 |
| *Tg*(*CMV*:*GFP-map1lc3b*) | GFP reporter transgenic zebrafish for Lc3 | 30 |
| *Tg*(*mpeg1:mCherryF*) | Membrane-localized mCherry reporter for zebrafish macrophages | 33 |
| *optn*^+/+^/GFP-Lc3 | Siblings of *optn*^∆5n/∆5n^ /GFP-Lc3 carrying a transgenic GFP-Lc3 reporter | In this study |
| *optn*^∆5n/∆5n^/GFP-Lc3 | *optn^ibl51^* mutant line carrying a transgenic GFP-Lc3 reporter | In this study |
| *p62*^+/+^/GFP-Lc3 | Siblings of *p62*^∆37n/∆37n^/GFP-Lc3 carrying a transgenic GFP-Lc3 reporter | In this study |
| *p62*^∆37n/∆37n^/GFP-Lc3 | *p62^ibl52^* mutant line carrying a transgenic GFP-Lc3 reporter | In this study |
| *optn*^∆5n/∆5n^ | *optn^ibl51^* mutant line | In this study |
| *p62*^∆37n/∆37n^ | *p62 ^ibl52^* mutant line | In this study |
