# Supplementary material for: The selective autophagy receptors Optineurin and p62 are both required for zebrafish host resistance to mycobacterial infection
Source: PLoS Pathog. 2019 Feb 28;15(2):e1007329. doi: 10.1371/journal.ppat.1007329 (PMC6413957; doi:10.1371/journal.ppat.1007329)
Supplement: S2 Table — (DOCX) [file ppat.1007329.s008.docx]

**S2 Table. Target sites for CRISPR/Cas 9 systems**

| Gene | Name | Target location | Sequence(5’-3’) |
| --- | --- | --- | --- |
| *optn* | *optn* target site | Exon 2 | GCTGGAAAAAAGTGGAGCTG |
| *p62* | *p62* target site | Exon 3 | GGACCAGGAGGGCTAAAGTG |
