# Supplementary material for: The selective autophagy receptors Optineurin and p62 are both required for zebrafish host resistance to mycobacterial infection
Source: PLoS Pathog. 2019 Feb 28;15(2):e1007329. doi: 10.1371/journal.ppat.1007329 (PMC6413957; doi:10.1371/journal.ppat.1007329)
Supplement: S3 Table — (DOCX) [file ppat.1007329.s009.docx]

**S3 Table. Primers for complementation and amplification of sgRNA**

| Name | Forward (5’-3’)* | Reverse (5’-3’) |
| --- | --- | --- |
| *optn* sgRNA template | GCG**TAATACGACTCACTATAG**GCT  GGAAAAAAGTGGAGCTGGTTTTAG  AGCTAGAAATAGCAAGTTAAAATA  AGGCTAGTC | GATCCGCACCGACTCGGTGCCACT  TTTTCAAGTTGATAACGGACTAGC  CTTATTTTAACTTGCTATTTCTAG  CTCTAAAAC |
| *p62*  sgRNA template | GCG**TAATACGACTCACTATAG**GGA  CCAGGAGGGCTAAAGTGGTTTTAG  AGCTAGAAATAGCAAGTTAAAATA  AGGCTAGTC |  |
| sg RNA amplify | GCGTAATACGACTCACTATAG | GATCCGCACCGACTCGGT |

* T7 promoter: **TAATACGACTCACTATAG**. The underlined sequence indicates the target sites for gRNAs designed to mutate *optn* or *p62*
