# Supplementary material for: The selective autophagy receptors Optineurin and p62 are both required for zebrafish host resistance to mycobacterial infection
Source: PLoS Pathog. 2019 Feb 28;15(2):e1007329. doi: 10.1371/journal.ppat.1007329 (PMC6413957; doi:10.1371/journal.ppat.1007329)
Supplement: S4 Table — (DOCX) [file ppat.1007329.s010.docx]

**S4 Table. MO sequences**

| Gene | Name | Target location | Sequence(5’-3’) |
| --- | --- | --- | --- |
| *optn* | *optn* MO | Intron2>Exon 2 | AGAGCCTCTGTGGGATGCATATAAT |
| *p62* | *p62* MO | Intron1>Exon 2 | CTTCATCTAGAGACAAAGTTCAGGA |
