# Supplementary material for: The selective autophagy receptors Optineurin and p62 are both required for zebrafish host resistance to mycobacterial infection
Source: PLoS Pathog. 2019 Feb 28;15(2):e1007329. doi: 10.1371/journal.ppat.1007329 (PMC6413957; doi:10.1371/journal.ppat.1007329)
Supplement: S5 Table — (DOCX) [file ppat.1007329.s011.docx]

**S5 Table. Primers used in this study**

| Gene | Type | Species | Accession | Forward (5’-3’) | Reverse (5’-3’) |
| --- | --- | --- | --- | --- | --- |
| *optn* | PCR-cDNA | ZF | ENSDART00000014036.10 | ATCAGGAAGAGCAGCATTTCCC | TTAATCTGAAACCCTCCAGACT |
| *p62* | PCR-cDNA | ZF | ENSDART00000140061.2 | GTCGGCTGAAGTAGGAAACG | ACCCTCCAGGTTTATGCTTG |
| *optn* | RT-PCR | ZF | ENSDART00000014036.10 | GGACATTAGTCACCCACGT | TTGGAGTTCAGAGTTCATCGCA |
| *p62* | RT-PCR | ZF | ENSDART00000140061.2 | ATTTGCAGCGAAAAGTGCTC | AGTGAACGGAAACCCAGGAA |
| *Optn* | Q-PCR | ZF | ENSDART00000014036.10 | GACTGAACACTATGGCGTGGA | GAATGCGAATCTGACCTCT |
| *p62* | Q-PCR | ZF | ENSDART00000140061.2 | GTCATATGGGTCCATCTCCAAT | AGGTGGGGCACAAGTCATAA |
| *il1b* | Q-PCR | ZF | ENSDART00000169225 | TGTGTGTTTGGGAATCTCCA | CTGATAAACCAACCGGGACA |
| *cxcl11aa* | Q-PCR | ZF | ENSDART00000169606 | ACTCAACATGGTGAAGCCAGTGCT | CTTCAGCGTGGCTATGACTTCCAT |
| *tnfa* | Q-PCR | ZF | ENSDART00000025847 | AGACCTTAGACTGGAGAGATGAC | CAAAGACACCTGGCTGTAGAC |
| *cxcl8a* | Q-PCR | ZF | ENSDART00000111598 | TGTGTTATTGTTTTCCTGGCATTTC | GCGACAGCGTGGATCTACAG |
| *ppaib* | Q-PCR | ZF | ENSDART00000166085 | ACACTGAAACACGGAGGCAAAG | CATCCACAACCTTCCCGAACAC |
| *optn* ∆LIR 1 | PCR | ZF | ENSDART00000014036.10 | GGAATTCGGATCAGGAAGAGCAGCATTTC | GGAGTTGCTAGGTGAACCTTGA |
| *optn* ∆LIR 2 | PCR | ZF | ENSDART00000014036.10 | AGAATAGCTGATGATGACTTA AAAGTG | AAGGCCTTTTAATCTGAAACCCTCCAGACTGAT |
| *optn* | PCR-genotyping | ZF | ENSDART00000014036.10 | AGTTTAGAGGAGACCCTCCAGC | AGAGGTCAGATTCTTCGCATTC |
| *p62* | PCR-genotyping | ZF | ENSDART00000140061.2 | CATCTTGGATTCATCATTACGTA | TCATATGGGGGGTCCTCCT |
