# Supplementary material for: The selective autophagy receptors Optineurin and p62 are both required for zebrafish host resistance to mycobacterial infection
Source: PLoS Pathog. 2019 Feb 28;15(2):e1007329. doi: 10.1371/journal.ppat.1007329 (PMC6413957; doi:10.1371/journal.ppat.1007329)
Supplement: S6 Table — (DOCX) [file ppat.1007329.s012.docx]

**S6 Table. Accession numbers of selective autophagy receptors**

| *Proteins*  *Species* | *Accession* | | | | |
| --- | --- | --- | --- | --- | --- |
|  | *Optineurin* | *P62* | *Calcoco2* | *TAXBP1* | *NBR1* |
| *Danio rerio* | ENSDARG00000002663 | ENSDARG00000075014 | ENSDARG00000052515 | ENSDARG00000098288 ENSDARG00000056856 | ENSDARG00000077297 ENSDARG00000078772 |
| *Homo sapiens* | ENSG00000123240 | ENSG00000161011 | ENSG00000136436 | ENSG00000106052 | ENSG00000188554 |
| *Pan troglodytes* | ENSPTRG00000002298 | ENSPTRG00000017626 | ENSPTRG00000009363 | ENSPTRG00000019025 | ENSPTRG00000009241 |
| *Mus musculus* | ENSMUSG00000026672 | ENSMUSG00000015837 | ENSMUSG00000006056 | ENSMUSG00000004535 | ENSMUSG00000017119 |
| *Xenopus tropicalis* | ENSGALG00000013738 | ENSGALG00000035804 | ENSGALG00000001525 | ENSGALG00000042822 | ENSPTRG00000009241 |
| *Gallus gallus* | ENSXETG00000009111 | ENSXETG00000015913 | ENSXETG00000022806 | ENSXETG00000000752 | ENSXETG00000014883 |
| *Takifugu rubripes* | ENSTRUG00000010419 | ENSTRUG00000017345 | ENSTRUG00000011902 | ENSTRUG00000018222 ENSTRUG00000015394 | ENSDARG00000078772 ENSDARG00000078772 |
